# Intelligent reprogramming of wheat for enhancement of fungal and nematode disease resistance using advanced molecular techniques

**DOI:** 10.3389/fpls.2023.1132699

**Published:** 2023-05-10

**Authors:** Muhammad Jabran, Muhammad Amjad Ali, Adil Zahoor, Ghulam Muhae-Ud-Din, Taiguo Liu, Wanquan Chen, Li Gao

**Affiliations:** ^1^ State Key Laboratory for Biology of Plant Diseases, Institute of Plant Protection, Chinese Academy of Agricultural Sciences, Beijing, China; ^2^ Department of Plant Pathology, University of Agriculture, Faisalabad, Pakistan; ^3^ Department of Biotechnology, Chonnam National University, Yeosu, Republic of Korea

**Keywords:** wheat diseases, marker assisted selection, quantitative trait locus, genome wide association studies, CRISPR/Cas-9 system

## Abstract

Wheat (*Triticum aestivum* L.) diseases are major factors responsible for substantial yield losses worldwide, which affect global food security. For a long time, plant breeders have been struggling to improve wheat resistance against major diseases by selection and conventional breeding techniques. Therefore, this review was conducted to shed light on various gaps in the available literature and to reveal the most promising criteria for disease resistance in wheat. However, novel techniques for molecular breeding in the past few decades have been very fruitful for developing broad-spectrum disease resistance and other important traits in wheat. Many types of molecular markers such as SCAR, RAPD, SSR, SSLP, RFLP, SNP, and DArT, etc., have been reported for resistance against wheat pathogens. This article summarizes various insightful molecular markers involved in wheat improvement for resistance to major diseases through diverse breeding programs. Moreover, this review highlights the applications of marker assisted selection (MAS), quantitative trait loci (QTL), genome wide association studies (GWAS) and the CRISPR/Cas-9 system for developing disease resistance against most important wheat diseases. We also reviewed all reported mapped QTLs for bunts, rusts, smuts, and nematode diseases of wheat. Furthermore, we have also proposed how the CRISPR/Cas-9 system and GWAS can assist breeders in the future for the genetic improvement of wheat. If these molecular approaches are used successfully in the future, they can be a significant step toward expanding food production in wheat crops.

## Introduction

Wheat (*Triticum aestivum* L.) is one of the most widely cultivated cereal grain crops and is a major source of calories for the world’s ever-increasing population (Gupta et al., 2020). Wheat yield enhanced significantly as a result of the green revolutions of the 1960s and 1980s, mainly in Southeast-Asia. In 2020-2021, global wheat production was 772.6 million tons (Nigus et al., 2022). However, current wheat production patterns do not give the impression of feeding the estimated population of nine billion people by 2050 (Curtis and Halford, 2014). The production of wheat grain per unit area must expand to meet increasing human demands, as expansion in the area under cultivation seems impossible due to urbanization and other problems, i.e., drought, water logging and salinity. Hence, significant work has been done in the past to increase the productivity of wheat worldwide after the green revolution. Despite this significant enhancement in wheat production, crop yield remains sensitive to several biotic and abiotic diseases (Jabran et al., 2021). Plant diseases are the key players affecting the yield of cereal food crops and have remained the main hindrance in achieving food security and safety (Montesclaros and Teng, 2021). Pests and pathogens damage 40–50% of crop yields. (Khan et al., 2021). A brief survey of the peer-reviewed literature showed that major wheat fungal diseases such as bunts, smuts, and rusts are difficult to manage due to the alternate existence of wheat pathogens on weeds or other host plants (Figueroa et al., 2018). Elimination of the fungus is difficult due to the ability of spores to remain viable for longer periods of time in the soil and air (Eversmeyer and Kramer, 2000). Similarly, the occurrence of diseases fluctuates greatly from season to season, depending on the conduciveness of environmental conditions, nature of pathogen, and host susceptibility (Jevtić et al., 2020). Furthermore, the intensive use of synthetic fertilizers over the years has taken a toll on the health of soil and has badly affected the environment as well as food quality. Fungal diseases such as rusts are extremely adaptive, and new races emerge quickly, infecting previously disease-resistant plants. Therefore, enhancement of plant resistance against the diseases caused by fungi, nematodes, viruses, and bacteria has great potential to increase crop production per unit area through resistance breeding approaches. These breeding systems were built on simple theories, which include identification of novel variation through sexual recombination and selecting offspring with the most desired traits. This all began as a visual selection, but as science progressed, it became convincing to utilize scientific data to select better plants for the development of disease resistant/tolerant crop varieties. The genetics of some types of plant defense against pathogens is simple and has been extensively studied using conventional phytopathology, breeding, and genetics approaches. Classical quantitative genetics has provided the tools for research related to complicated disease resistance in the past. However, quantitative genetics deals with the inheritance of complex traits in plant populations. In the context of plant disease resistance, it involves the use of statistical methods to analyze the genetic basis of resistance to various diseases (Fischer et al., 2004). Furthermore, conventional wheat breeding is a process to control major diseases in wheat by selecting and developing disease-resistant varieties through crossbreeding and hybridization. The main diseases are rusts, smuts, and leaf and stem diseases, and breeding for resistance involves selecting for plants with resistance genes to overcome the disease. This is done by identifying resistant plants and crossing them with other plants to create new varieties with the desired traits. The development of leaf rust-resistant, powdery mildew-resistant, and FHB-resistant cultivars is done by crossing wheat varieties with natural resistance and selecting for the most resistant progeny. However, this process can take multiple years and generations to produce a cultivar with the desired level of resistance.

To address and manage these biotic issues, molecular marker approaches are a breakthrough genomic tool for producing disease-resistant germplasm with high production (Mores et al., 2021). Likewise, new molecular approaches must be included in plant breeding programs to enhance resistance against wheat diseases (Ali et al., 2019). Many researchers have explored the potential of molecular markers for enhancing the speed and proficiency of breeding program (Bhanu et al., 2016), and there is a large body of literature available on the topic of how to use markers in breeding assessment to support a variety of tasks such as trait mapping, germplasm evaluation, and cultivar identification. Similarly, markers can be used to identify the genetic loci that control traits of interest such as disease resistance, yield, or seed size. This information can then be used to select plants with desirable traits for breeding purposes (Collard et al., 2005). Therefore, marker-assisted selection (MAS) has been established as a promising breeding strategy using molecular markers. Conventional simple sequence repeats (SSRs) have remained the most prevalent technology utilized by the public sector in breeding programs. They have been applied in cross-population meta-analyses (Swamy and Sarla, 2008), allelic diversity evaluations and biparental mapping research such as quantitative trait loci (QTL) mapping and fine-mapping (Yadaw et al., 2013). QTL mapping depends upon the application of DNA markers to identify genomic regions associated with complicated and polygenic types of disease resistance (Young, 1996). Furthermore, novel pathogen mutations frequently cause a higher risk to crops. For instance, Ug99 is the most virulent fungal strain of wheat stem rust disease (Singh et al., 2011), and wheat blast (Ceresini et al., 2018) severely harms wheat productivity in different countries. These ever-mutating pathogens have diverted the attention of plant breeders to constantly seek novel resistance gene for the management of crop disease. To combat this problem, partial/polygenic resistance has been defined as more durable and broad-spectrum resistance (BSR) against multiple pathogen species/strains or races that is modulated by QTLs (Li S. et al., 2021).

**Figure 1:**
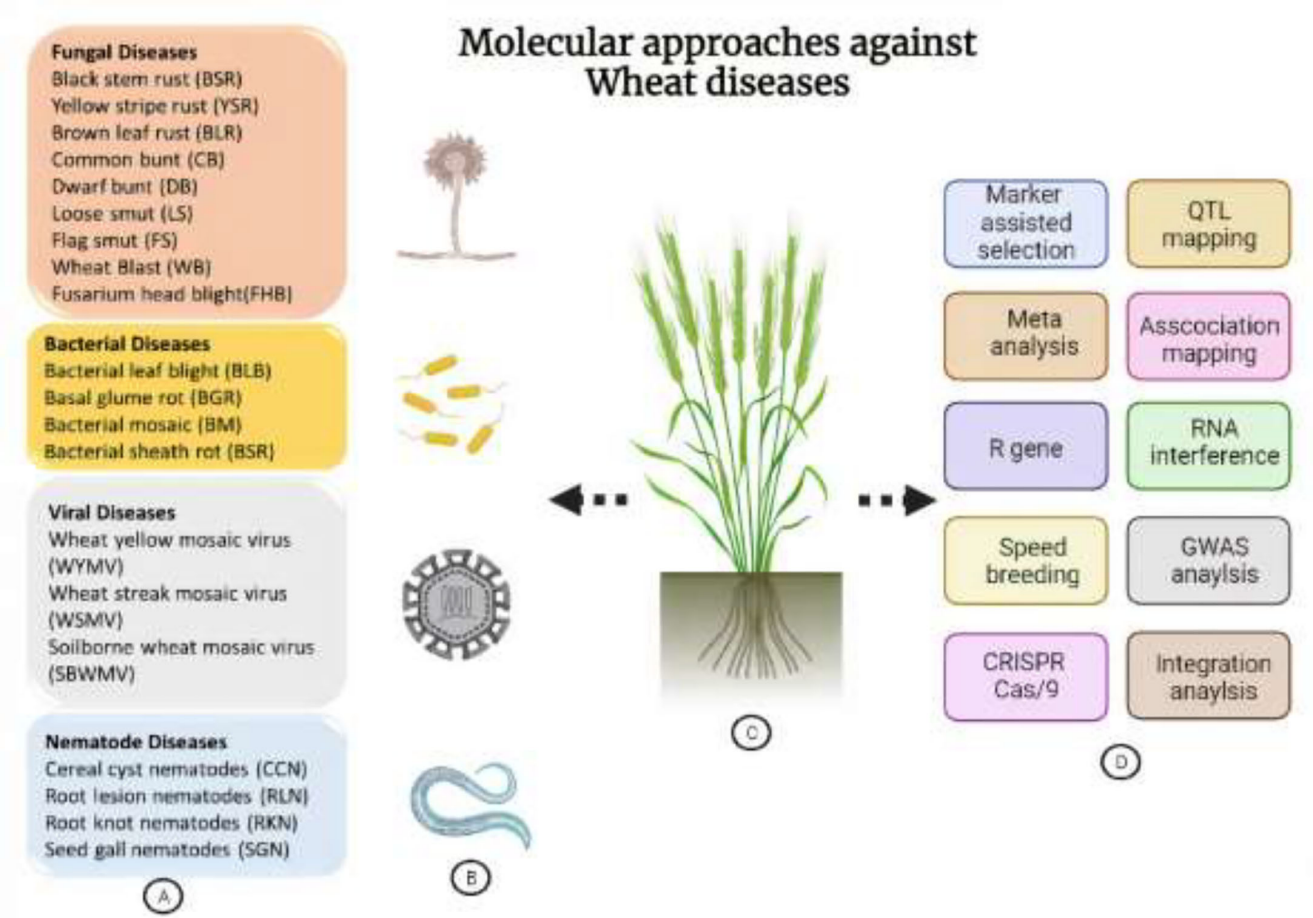
Overview of destructive wheat diseases and molecular approaches to develop resistance against them. **(A)** Categorical description of wheat pathogens. **(B)** Schematic representation of different classes of pathogens. **(C)** A wheat plant demonstrating its different pathogens and approaches. **(D)** Representation of various genome-editing, genomics, conventional breeding-based techniques and being employed for the execution of wheat immunity against pathogens.

Most of the modern MAS research has concentrated on novel single nucleotide polymorphism (SNP) technologies following recent advancements in next-generation sequencing that reveal the entire genomes of crop plants for SNP identification (Liu et al., 2022). Genotyping by sequencing followed by SNP identification is a high throughput genomics approach that has been largely used for genome-wide association studies (GWAS) in relation to disease resistance in crop plants (Begum et al., 2015). Similarly, genome editing technology alters a specific sequence of DNA in the genome. A restriction enzyme that can identify the specific sequences in the host genome is required for genome editing. These enzymes act like molecular scissors that not only locate the genomic position but also cut specific sequences (Khalil, 2020). Among genome editing tools, an efficient and easy technique is the CRISPR/Cas9-system multiplex genome editing technique, which has been particularly important for understanding the complicated features of wheat disease resistance (Zhang et al., 2021). Using the CRISPR/Cas9 system, mutations in host susceptibility (S) genes provided broad-spectrum disease resistance against plant pathogens (Ahmad et al., 2020). Crop disease resistance and/or tolerance to major biotic (e.g., bacterial blight of rice and rice blast) and abiotic stresses (e.g., drought and salinity) have both been improved using CRISPR-based genome editing (Jaganathan et al., 2018). Likewise, (Baltes et al., 2015) illustrated the use of CRISPR/Cas9 technology to generate *geminivirus* resistant plants, and this technology has great potential for improving disease resistance in wheat. Furthermore, molecular technology has played an important role in the development of disease-resistant wheat cultivars. Scientists around the world have worked to combat various diseases affecting wheat crops such as Fusarium head blight, *Septoria tritici* blotch, leaf rust, and stem rust. By introducing genes that confer resistance to these diseases, they have developed cultivars that are now widely grown by farmers, such as “Avocet R” for FHB resistance, “Condor” for STB resistance, “Zambezi” for leaf rust resistance, and “Moleleka” for stem rust resistance. These efforts aim to provide farmers with crops that are more productive and sustainable, contributing to the overall growth of the agriculture industry (McLaughlin et al., 2021). In this review, we provide a detailed and comprehensive account of research advancements in QTL mapping, GWAS and genome editing technologies for the enhancement of resistance against wheat bunts, rusts, smuts, nematodes, and other important diseases. We have further reviewed most of the QTLs identified and found to be associated with resistance against major diseases of wheat. The general idea of molecular approaches to develop resistance against major wheat diseases is shown in **Figure 1**.

## Marker assisted selection of wheat resistance

Marker Assisted Selection (MAS) of wheat resistance is a plant breeding technique that uses molecular markers to select for resistance to specific diseases. This technique combines traditional plant breeding practices with modern molecular biology tools. In MAS, molecular markers that are associated with a particular trait of interest (such as disease resistance) are identified and used to screen a large number of wheat varieties. This allows plant breeders to identify individuals with the desired trait more efficiently and speeds up the breeding process by reducing the amount of time required to test each variety in the field (Francia et al., 2005). The most broadly utilized application of DNA markers is marker-assisted selection (MAS), which is a type of indirect selection. Once plant physical attributes have been mapped, a closely related DNA marker can be used to screen many samples for progeny with appropriate characteristics (Miedaner and Korzun, 2012). Advances in molecular genetics have led to the invention of DNA tags and marker assisted selection procedures for resistant cultivars (Collard and Mackill, 2008). The introduction of marker-assisted molecular breeding in the context of worldwide climate change and water constraints in arid and semiarid countries coupled with expanding global populations is a prerequisite for crop improvement (Hickey et al., 2019). Molecular markers are obviously not impacted by environmental factors and unchanged by plant developmental stages and can be detected at any stage of plant growth. MAS has become accessible for qualities regulated by most important genes along with quantitative trait loci (QTLs) and ease of use of molecular markers and genetic maps (Hasan et al., 2021). Sequence Characterized Amplified Region (SCAR) (Gold et al., 1999), Simple-sequence repeats SSRs (Wang et al., 2002), random amplified polymorphic DNA (RAPD) (Qi et al., 1996), microsatellites or simple sequence length polymorphisms (SSLPs) (Ma et al., 2001), restriction fragment length polymorphism (RFLP) (Metakovsky et al., 2021) and novel single-nucleotide polymorphism, (SNP) (Wu et al., 2018) are the different molecular markers that have been reported for the molecular identification of plant pathogens and widely used in plant breeding (Thomson, 2014). Hence, MAS could be useful in helping traditional plant breeding procedures in selecting phenotypic features for disease resistance screening (Todorovska et al., 2009). Accordingly, conventional plant breeding approaches, as well as functional genetic equipment and existing molecular markers (Gupta et al., 2010), can assist a breeder in producing advanced wheat genotypes that are resistant to fungal pathogens in order to reduce production losses. Hence, high costs in MAS that requires specialized laboratory equipment and skilled personnel, which can be expensive. The cost of the molecular markers, DNA extraction, and genotyping can also add to the cost of MAS. Any errors in any of these steps can impact the accuracy of the results.

## Quantitative trait loci for plant disease resistance in wheat

**Figure 2:**
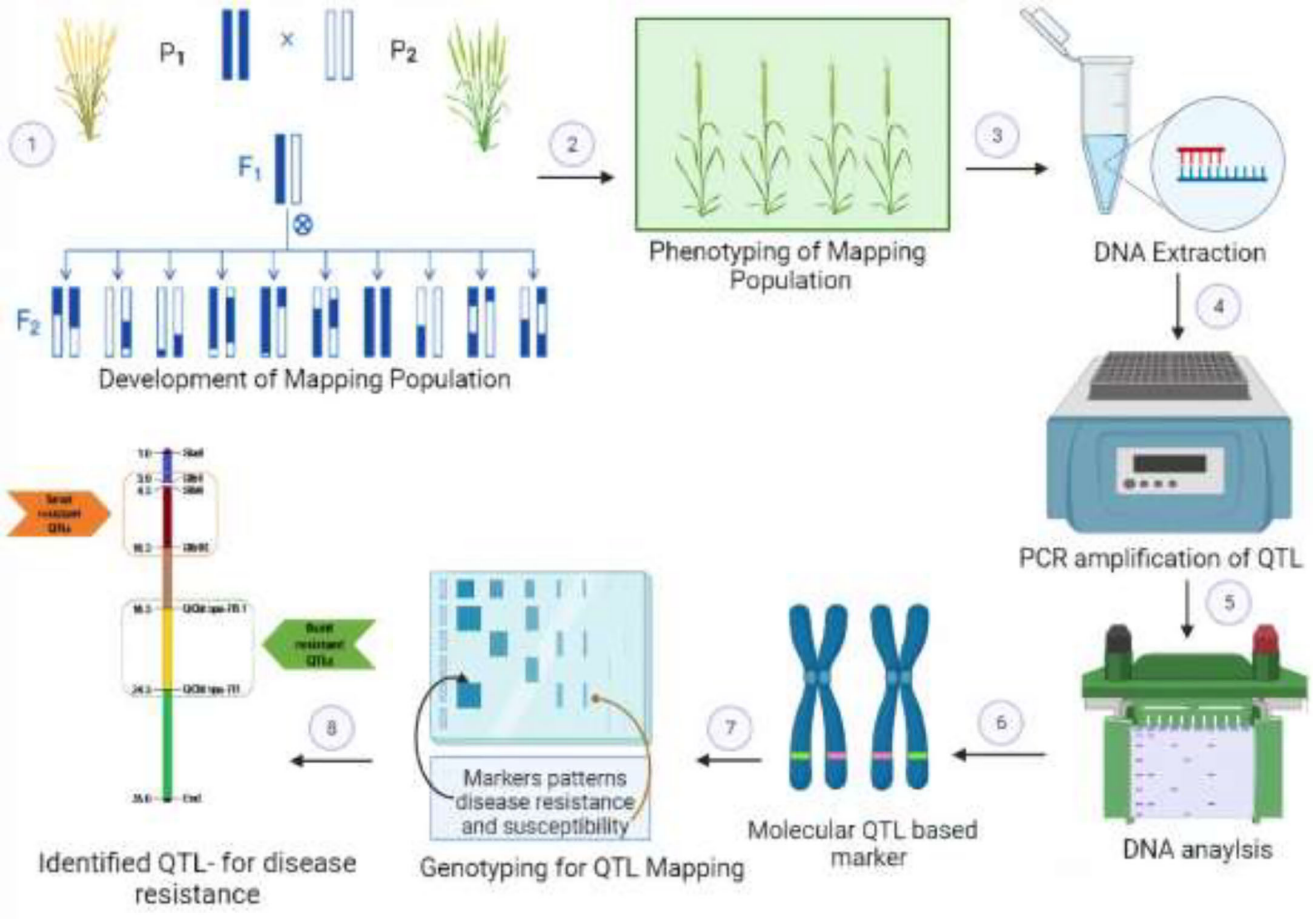
Scheme of marker-assisted selection (MAS) and identification of quantitative trait loci (QTLs) involved in wheat disease resistance. 1: Biparental mapping population development, i.e., F_2_ plants, NILs, RILs, BILs, etc., segregating for disease resistance. 2: Precise phenotyping for resistance scaling to a specific pathogen. 3: Genomic DNA isolation from selected plants. 4-6: Evaluation of genome-wide distributed polymorphic DNA markers and construction of linkage maps. 7, 8: Statistical modeling of linkage groups using phenotypic-genotypic data followed by mapping of resistance-associated QTLs.

QTLs (quantitative trait loci) and genes are related, but they are not the same thing. A gene is a segment of DNA that codes for a specific protein or RNA. Each gene contains the information needed to make one or more specific proteins, and these proteins determine the traits of a plant, such as height and susceptibility to certain plant diseases. While QTLs are specific locations on a chromosome that are associated with a particular trait and often influenced by multiple genes. They are used to map and study the genetic basis of complex traits in plants. The identification of QTLs can provide insights into the genetic basis of a trait and can be used to improve breeding programs for disease resistance in plants (Vinod, 2009). QTLs are mapped by the identification of molecular markers that are linked with a certain trait. QTL mapping mainly involves the crossing of contrasting parents (resistant and susceptible) to develop the mapping population. This mapping population is subjected to the measurement of disease response, i.e., phenotyping, and is assessed for its genetic makeup through genotyping, i.e., amplification of DNA markers. The whole process of QTL mapping is shown in **Figure 2**.

Many quantitative plant attributes are regulated by several genes. With the rapid improvement of molecular technology, it is feasible today to map significant QTLs on chromosomes using molecular marker information (Phipps et al., 2022). Various statistical techniques have been developed for investigating mapping data and searching for important QTLs in crop plants. It is possible to select for a resistance gene without exposing the plant to the pathogen, disease, or harmful substance if the QTLs associated with that resistance are known in the same crop species. Successful association between a molecular marker and a QTL makes the selection more effective, reliable, and faster (Reynolds and Borlaug, 2006). Resistance to wheat diseases such as dwarf bunt, leaf rust, stripe rust, stem rust, and spot blotch is often controlled by one to three or more genes that can be identified with several DNA markers, which are particularly based on PCR technology (Randhawa et al., 2013). Seed storage protein loci, genome identification, protein sequences, resistance gene positions, intrachromosomal mapping of genes for dwarfing and vernalization, characterization of genes and QTLs controlling tissue culture response, resistance against nematodes, and production level have all been mapped using molecular markers in wheat crops. There are various kinds of molecular markers that have their own set of benefits and drawbacks. However, most scientists are now aware of the limitations of RAPDs and other molecular markers with less frequent use. The amplified fragment length polymorphism (AFLP) technique uses PCR to selectively amplify DNA segments that have been processed with one or two restriction enzymes (Amom and Nongdam, 2017). Soon after, microsatellite markers, also known as SSRs (simple sequence repeats), were developed to replace RAPD and RFLP (Harshitha and Sandal, 2022). SSRs have been the most developed and used DNA markers for the characteristics associated with disease resistance in crop plants, especially wheat. For instance, several nematode resistance QTLs based on SSR markers have been previously reviewed (Seid et al., 2021). SNP data are widely utilized to detect marker-trait relationships in quantitative trait locus (QTL) mapping research (Rajesh et al., 2021). A large number of QTLs associated with resistance to important diseases have been identified and mapped on different chromosomes of wheat. However, it also has some limitations such as QTL analysis can only identify the general location of the genes associated with a particular trait, but not the specific genes themselves. This can make it difficult to identify the exact genes responsible for disease resistance. The detailed accounts of these mapped QTLs associated with bunts, rusts, smuts, and wheat parasitic nematodes are given below.

## Mapped QTLs for bunt diseases

Dwarf bunt (*Tilletia controversa* J.G. Kühn) and common bunt (*T. caries* and *T. foetida*) are important global quarantine diseases that decrease the quality and quantity of wheat grains (Gao et al., 2014). Teliospore germination and the development of both pathogens on wheat plants are favored by continuous snow cover in the field (Wilcoxson, 1996; Rush et al., 2005). These seed and soil-borne diseases are usually sufficient to produce unpleasant flour odors that reduce grain quality and cause yield losses of more than 80% (Castlebury et al., 2005). However, dwarf and common bunt fungal diseases are difficult to characterize due to their similar morphology and ecology (Li et al., 2018). Bunt diseases of wheat can effectively be controlled by different measures, such as systemic fungicides, e.g., difenoconazole. Hence, most scientists’ interest is diverted to genetic resistance to bunt diseases due to attention to organic farming and a focus on more sustainable agricultural production practices. Recent research has attempted to find and map host resistance by using a gene mapping strategy (Zou et al., 2017) and genome-wide analysis (Mourad et al., 2018). Several QTLs have been identified in wheat that are associated with bunt resistance. Similarly, some molecular markers have also been developed for pathogen identification in bunt diseases. Sixteen resistance genes (*Bt1–Bt15*) and BtP have been listed, and several resistance sources are accessible for bunt of wheat (Muellner et al., 2020).

For instance, a specific SCAR marker was developed to detect *Tilletia foetida* (Zhang et al., 2012). Therefore, the application of molecular approaches for the development of bunt-resistant germplasm is essential in many wheat-growing countries, mainly where organic wheat is farmed. Few molecular markers have been discovered to be associated with bunt resistance (Wang et al., 2019). For instance, (Fofana et al., 2008) mapped QTLs associated with resistance to common bunt in a doubled haploid population derived from the cross of two spring wheat accessions RL4452 x AC Domain. They found one QTL on 1BS that was linked to common bunt disease. Furthermore, QTLs were discovered on chromosomes 7A (Dumalasová et al., 2012) and 7B (Knox et al., 2013). Earlier, the genes present in breeding lines were identified and germplasm was based on their response to certain pathogen strains. These pathogen strains have been discovered based on their virulence in the current collection of differential lines. There is much evidence that the current set of differential lines is not always monogenic for bunt pathogenic races. Race D-18 showed minimal virulence toward the Bt8 differential line, but races L-18 and D-19 were virulent toward the *Bt8* differential line. Similarly, the D-7 strain is not virulent against the *Bt8* line, while it is virulent against CI 9342 and PI 636146, which are thought to contain Bt8 based on their responses to L-18, D-18, and D-19 (Goates, 2012). *Bt8* gene is highly resistant to dwarf bunt disease and developed from wheat cultivar PI 178383 in the United States. *Bt8* gene is a well-known gene from cultivars that does not cooperate with known strains of dwarf bunt disease in the United States (Goates, 2012). Similarly, amplified fragment length polymorphism (AFLP) was used to examine a total of 30 primer combinations for detecting DNA polymorphisms in *T. controversa* and related species. According to preliminary findings, the Bt11 gene is found on chromosome 3B and may be linked to QTLs such as Xbarc180, Xwmc623, Xwmc808, and Xgwm285. By employing 1R-specific markers, the bunt resistance gene introduced from Triticale (Line F00628G34-1- containing a 1A/1R translocation) may lead to successful MAS (Ciucă, 2011). SYBR Green I and TaqMan real-time polymorphic chain reaction analyses were established to identify TCK with a finding limit of 0.1 fg (Gao et al., 2014). Likewise, the Diversity Arrays Technology (DArT) and Illumina Infinium 9K iSelect marker platforms were used to genotype the population. Three Utah State University (USU) experiments discovered a QTL Q. ui-1A on 1A that explained 11–15% of phenotypic variation for resistance to dwarf bunt in recombinant inbred line (IDO444) of wheat (Chen et al., 2016). Likewise, Diversity Arrays Technology (DArT) and Illumina Infinium 9K iSelect marker platforms were used to genotype the population. Three Utah State University (USU) experiments discovered a QTL Q.ui-1A on 1A that explained 11–15% of phenotypic variation for resistance to dwarf bunt in a recombinant inbred line (IDO444) of wheat [63]. Likewise, the study was undertaken for DB resistance across four growing seasons in a field nursery in Logan, Utah. The Illumina 90 K SNP iSelect marker platform was used to genotype the population. On chromosomes 6DL and 7AL, the two most important QTLs were recognized (Q.DB.ui-6DL) and (Q.DB.ui-7AL), respectively. Comparative research proposed that Q.DB.ui-6DL was found in the same location as the Bt9 gene of CB resistance, and Q.DB.ui-7AL was discovered at a new bunt resistance locus. Both resistance QTLs were mapped against bunt disease resistance in the wheat line ‘IDO835’ in gene-rich (NBS-LRR and kinase genes) areas using the Chinese Spring reference sequence and annotations (Wang et al., 2019). An inter simple sequence repeat (ISSR) molecular marker was reported for the first time by (Yao et al., 2019) and is a quick diagnostic approach for the detection of teliospores of *Tilletia laevis* Kühn. Similarly, SYBR Green I real-time PCR and sequence characterized amplified region (SCAR) is fast and highly specific for the identification of the teliospores of *T. laevis* depending upon the ISSR tool. This technique enables efficient and correct differentiation among pathogens, particularly the pathogens *T. tritici* and *T. controversa*, which are very similar (Li et al., 2018). Similarly, dwarfism and common QTLs for bunt resistance were found on chromosomes 1AL, 1BS, 7AL, and 7DS. Kompetitive allele-specific PCR (KASP) was successfully employed for QTL validation. These KASP markers have the potential to aid targeted QTL introgression into superior wheat germplasm and speed up bunt resistance breeding. The combination of multiple resistance genes in the same genetic background can provide long-term protection against both common and dwarf bunts (Muellner et al., 2021). Various QTLs associated with bunt resistance are given in **Table 1**.

**Table 1:** QTLs reported to be associated with bunt disease resistance genes in wheat, their origin, chromosomal positioning and linked molecular markers.

Sr. No.	QTLs	Origin	Position	A. M. C	Source
**1**	Q.DB.ui-7DS	(RIL) Idaho 444	7DS	DArT marker	(Chen et al., 2016)
**2**	Bt12	(RIL) PI119333	7DS	SNP (KASP)	(Muellner et al., 2020)
**3**	Bt10	*T. aestivum*	6D	microsatellite markers	(Menzies et al., 2006)
**4**	Bt9	*T. aestivum*	6DL	SSR	(Steffan et al., 2017)
**5**	Bt1	*T. aestivum*	2B		(Al-Maaroof et al., 2016)
**6**	Bt4	*T. aestivum*	1B		(Briggs, 1933)
**7**	QCbt.crc-1B.1	*T. aestivum*	1BS	SSR	(Fofana et al., 2008)
**8**	QCbt.crc-1B.2	*T. aestivum*	1BL	SSR	(Fofana et al., 2008)
**9**	QCbt.spa-1B	*T. aestivum*	1B	microsatellite and DArT	(Singh et al., 2016)
**10**	QCbt.spa-4B	*T. aestivum*	4B	microsatellite and DArT	(Singh et al., 2016)
**11**	QCbt.spa-4D	*T. aestivum*	4D	microsatellite and DArT	(Singh et al., 2016)
**12**	QCbt.spa-7B.1	*T. aestivum*	7B	SSR	(Knox et al., 2013)
**13**	QCbt.spa-7D	*T. aestivum*	7D	microsatellite and DArT	(Singh et al., 2016)
**14**	Bt11	*T. aestivum*	3B	Xwmc808, Xgwm285	(Ciucă, 2011)
**15**	Bt13				(Dumalasová et al., 2012)

## Mapped QTLs for rust diseases

Wheat rust pathogens are host specific and macrocyclic. Leaf rust, stem rust, and stripe rust are important diseases of wheat worldwide (Sinha and Chen, 2021). Wheat rusts are caused by obligate, biotrophic fungal pathogens belonging to the family *Puccinacae* that can spread thousands of miles by wind and cause significant economic losses around the globe (Kolmer, 2013). For instance, only yellow or stripe rust of wheat causes up to 100% yield losses to susceptible varieties (Chen, 2005).

Additionally, (Park et al., 2011) proved the value of genetic resistance in the management of rust pathogens in their studies. The availability of various resistance genes is a requirement for producing cultivars with long-term rust resistance. Rust infection has become more common in the recent years, producing epidemics in wheat-growing areas across the United States and world (Jamil et al., 2020). This is mainly caused by substation changes in climatic conditions, which lead the pathogen to alter its genetic makeup and break resistance in the host. Ug99, a novel strain of stem rust, was discovered in Uganda in 1999, and its variants are pathogenic to approximately 80–90% of wheat cultivars and germplasm (Singh et al., 2011; Bhardwaj et al., 2014). Stripe rust has also appeared as the most harmful grain rust in several parts of the globe caused by *Puccinia striiformis* (Wellings, 2011). Spring wheat landrace PI 480035 proved highly resistant against stripe rust (Sthapit et al., 2014). In 2013 and 2014, the population was assessed in the research area, and seedling responses against three pathogen races (PSTv-14, PSTv-37, and PSTv-40) were tested under a controlled environment (Wan et al., 2017). Genotyping by sequencing and microsatellite markers were used to genotype the population within the entire wheat genome. On chromosome 1B, an important QTL (QYr.wrsggl1-1BS) was discovered. The nearest molecular markers included Xgwm273, Xgwm11, and Xbarc187 1.01 cM distal to QYr.wrsggl1-1BS, Xcfd59 0.59 cM proximal to QYr.wrsggl1-1BS, and XA365 3.19 cM proximal to QYr.wrsggl1-1BS, respectively (Sthapit Kandel et al., 2017). On 3B, a different QTL (QYr.wrsggl1-3B) was detected that was only significant for PSTv-40 and not in the field, showing that it negotiates race-specific resistance. When compared to markers linked with earlier studied Yr genes on 1B (Yr64, Yr65, and YrH52), QYr.wrsggl1-1BS appears to be a new resistance gene to yellow stripe rust. This novel gene can be adapted into modern breeding systems and resistance genes of adult plants to create cultivars with long-lasting resistance (Sthapit Kandel et al., 2017). The Yr15 gene has been finely mapped to a 0.77 cM area using BSR-Seq, which confers resistance to yellow rust in wheat, allowing the creation of effective MAS markers (Ramirez-Gonzalez et al., 2015). In diploid wheat species of both common wheat and durum wheat, more than 65 leaf rust resistance (Lr) genes have been reported through different molecular techniques (Bansal et al., 2008). The wild wheat relative *Aegilops tauschii* has been shown to contain most of these genes (Hiebert et al., 2007). In a study using the IWGSC RefSeq v2.0 physical map, 82 QTLs were mapped in two hexaploid wheat populations for resistance to leaf spot, leaf rust, stripe rust, and common bunt. 29 QTLs were associated with all combined environments, and 14 were stable across most environments. Ten chromosome arms had QTL clusters for resistance to 2-4 diseases, providing a resource for comparing QTLs in different populations based on physical information of all markers. A total of 82 QTLs related to resistance against Yr (36), Ls (18), Lr (15), and Cbt (13) were discovered by analyzing both individual and combined data from all environments (Iqbal et al., 2023).

The study led to determine the frequency of Iranian Pt races causes leaf rust of wheat, their virulence to key resistance genes and map quantitative trait loci (QTL) for resistance to different Pt races from 185 globally diverse wheat genotypes. Results showed that some Pt races were relatively frequent in Iran and that certain resistant genes were still effective against the pathogen population. Genome-wide association study (GWAS) resulted in the identification of 62 significant marker-trait associations (MTAs) belonged to 34 QTLs across 16 chromosomes. The known and novel QTLs associated with different Pt races can be used in future wheat breeding programs for durable resistance (Talebi et al., 2023). Furthermore, a study evaluated 441 synthetic hexaploid wheat (SHW) accessions for resistance to spot blotch (SB) caused by *Bipolaris sorokiniana*. The panel showed high resistance, with 250 accessions being resistant and 161 showing moderate resistance. A genome-wide association study (GWAS) revealed 41 significant marker-trait associations for SB resistance, located on different chromosomes, but none had a major effect. This is the first GWAS to identify markers and resistant SHW lines for SB resistance in wheat breeding (Haugrud et al., 2023). Various QTLs linked to rust resistance are given in **Table 2**.

**Table 2:** QTLs reported to be associated with rust disease resistance genes in wheat, their origin, chromosomal positioning and linked molecular markers.

Sr. No.	QTLs	Origin	Position	A. M. C	Source
Leaf rust (Puccinia triticina)					
**1**	*Lr1*	*T. aestivum*	7BL	RFLP/STS	(Akın et al., 2013)
**2**	*Lr3*	*T. aestivum*	6BL	RFLP	(Herrera-Foessel et al., 2007)
**3**	*Lr9*	*Aegilops* *umbellulata*	6B	RAPD/STS, RFLP	(Autrique et al., 1995)
**4**	*Lr10*	*T. aestivum*	1AS	RFLP/STS, STS	(Feuillet et al., 2003)
**5**	*Lr12*	*T. aestivum*	4B	SSR	(Singh and Bowden, 2011)
**6**	*Lr13*	*T. aestivum*	2BS	RFLP, SSR	(Bansal et al., 2008)
**7**	*Lr14*	*T. aestivum*	7BL	SSR	(Herrera-Foessel et al., 2007)
**8**	*Lr14a*	*T. durum*	7BL	SNP	(Terracciano et al., 2013)
**9**	*Lr15*	*T. aestivum*	2DS	SSR	(Dholakia et al., 2013)
**10**	*Lr16*	*T. aestivum*	2BS	SSR	(Kassa et al., 2017)
**11**	*Lr19*	*Agropyron elongatum*	7DL	STS, RAPD/SSR	(Zhang et al., 2011)
**12**	*Lr20*	*T. aestivum*	7AL	RFLP	(Neu et al., 2002)
**14**	*Lr21*	*T. tauschii*	1DS	RFLP; KASPar	(Huang and Gill, 2001)
**15**	*Lr22*	*T. tauschii*	2DS	SSR	(Hiebert et al., 2007)
**16**	*Lr23*	*T. turgidum*	2BS	RFLP	(Nelson et al., 1997)
**17**	*Lr24*	*Agropyron elongatum*	3D	RFLP, RAPD/STS, SCAR	(Gupta et al., 2006)
**18**	*Lr25*	*S. cereale*	4BS	RAPD/SSR	(Singh et al., 2012)
**19**	*Lr26*	*Secale cereale*	1B	SCAR, SSR	(Zhou Y. et al., 2014)
**20**	*Lr27*	*T. aestivum*	3BS	RFLP, SSR	(Aravindh et al., 2020)
**21**	*Lr28*	*T. aestivum*	4AL	STS, SCAR	(Sohail et al., 2014)
**22**	*Lr29*	*Agropyron elongatum*	7DS	RAPD	(Vanzetti et al., 2011)
**23**	*Lr31*	*T. aestivum*	4BL	RFLP, SSR	(Nelson et al., 1997)
**24**	*Lr32*	*T. tauschii*	3DS	RFLP	(Autrique et al., 1995)
**25**	*Lr34*	*T. aestivum*	7DS	STS	(Dakouri et al., 2010)
**26**	*Lr35*	*A. speltoides*, *T. speltoides*	2BL	SCAR, STS	(Gold et al., 1999)
**27**	*Lr37*	*A. ventricosa*	2AS	STS/CAPS, ISSR	(Bulos et al., 2006)
**28**	*Lr38*	*Thinopyrum* *intermedium*	2AS	SSR	(Mebrate et al., 2008)
**29**	*Lr39*	*T. Tauschii*	2DS	SSR	(Raupp et al., 2001)
**30**	*Lr41*	*T. Tauschii*	1D		(Sun et al., 2009)
**31**	*Lr45*	*T. aestivum*	2A	AFLP, SSR	(Naik et al., 2015)
**32**	*Lr46*	*T. aestivum*	1BL	STS	(Mateos-Hernandez et al., 2006)
**33**	*Lr47*	*T. speltoides*	7AS	RFLP, CAPS	(Helguera et al., 2000)
**34**	*Lr48*	*T. aestivum*	2BS	SSR	(Bansal et al., 2008)
**35**	*Lr49*	*T. aestivum*	2AS, 2BL	SSR	(Bansal et al., 2008)
**36**	*Lr50*	*T. timopheevii*	2BL	SSR	(Brown-Guedira et al., 2003)
**37**	*Lr51*	*T. speltoides*	1BL	STS	(Helguera et al., 2005)
**38**	*Lr52*	*T. aestivum*	5BS	STS	(Bansal et al., 2011)
**39**	*Lr 58*	*T. aestivum*	Ncw1	SSR	(Kuraparthy et al., 2007)
**40**	*Lr60*	*T. aestivum*	1DS	SSR	(Hiebert et al., 2008)
**41**	*Lr63*	*T. monococcum*	3AS	SSR	(Kolmer et al., 2010)
**42**	*Lr64*	*T. dicoccoides*	1DS	SSR	(Kolmer et al., 2019)
**43**	*Lr67*	*T. aestivum*	4D	SSR	(Hiebert et al., 2010)
**44**	*Lr68*	*T. aestivum*	7BL	SSR, CAPS	(Herrera-Foessel et al., 2012)
**45**	*LrM*	*Aegilops* *markgraffii*	2AS	SNP based PCR markers	(Rani et al., 2020)
**46**	*TaCN-R with* *Lr13*	leaf rust in wheat	QLr.hnau-2BS	Linkage mapping	(Hou W. et al., 2023)
Stem rust (Puccinia graminis f. sp. tritici., P. recondita)					
**1**	*Sr2*	*T. turgidum*	3BS	STS, CAPS	(Mago et al., 2014)
**2**	*Sr9a*	*T. aestivum*	2BL	SSR	(Tsilo et al., 2007)
**3**	*Sr22*	*T. monococcum*	7AL	RFLP	(Periyannan et al., 2011)
**4**	*Sr24*	*Agropyron elongatum*	3DL	STS	(Mago et al., 2005)
**5**	*Sr25*	*Thinopyrum ponticum*	7BL	STS	(Liu et al., 2010)
**6**	*Sr26*	*Agropyron elongatum*,	6AL	STS	(Mago et al., 2005)
**7**	*Sr28*	*T. aestivum*	2BL	PCR	(Rouse et al., 2012)
**8**	*Sr35*	*T. aestivum*	3AL	SSR	(Zhang et al., 2010)
**9**	*Sr38*	*A. ventricosa*	2AS	STS/CAPS	(Helguera et al., 2003)
**10**	*Sr39*	*A. speltoides*	2B	STS	(Yu et al., 2014)
**11**	*Sr36*	*T. timopheevi*	2BS	SSR	(Yu et al., 2014)
**12**	*Sr47*	*Aegilops speltoides*	2B	SSR	(Klindworth et al., 2017)
**13**	*Sr52*	*D. villosum*	6AL	STS	(Yu et al., 2014)
**14**	*Sr R*	*Secale cereale*	1D	STS	(Mago et al., 2002)
**15**	*Sr32*	*A. speltoides*	2DS	SSR	(Mago et al., 2013)
**16**	*Sr43*	*T. aestivum*	7D	SSR	(Niu et al., 2014)
**17**	*Sr45*	*T. aestivum*	1DS	SSR/AFLP	(Periyannan et al., 2014)
**18**	*Sr54*	*Ae. tauschii*	2DL	SSR	(Ghazvini et al., 2013)
**19**	*Sr56*	*T. aestivum*	5BL	STS and SSR	(Bansal et al., 2014)
**20**	*QSr.dms-2B*	Stem rust of wheat	2B	SNPs	(Ciechanowska et al., 2022)
Stripe rust – (Puccinia striiformis)					
**1**	*Yr5*	*T. spelta*	2BL	STS	(Yan et al., 2003)
**2**	*Yr10*	*T. aestivum*	1BS	SSR, STS	(Yuan et al., 2018)
**3**	*Yr15*	*T. dicoccoides*	1BS	SSR	(Sun et al., 1997)
**4**	*Yr17*	*A. ventricosa*	2AS	STS/CAPS, SCAR	(Robert et al., 1999)
**5**	*Yr26*	*H. villosa*,	1B	SSR, EST-STS	(Wang et al., 2008)
**6**	*Yr28*	*T. aestivum*	4DS	RFLP	(Zheng et al., 2020)
**7**	*Yr50*	*T. aestivum*	4BL	SSR	(Liu et al., 2013)
**8**	*Yr51*	*T. aestivum*	4AL	DArT (Marker sun104)	(Randhawa et al., 2014)
**9**	*YrH52*	*T. dicoccoides*	1B	SSR	(Yan et al., 2011)
**10**	*Yr53*	*T. aestivum*	2B	RGAP/SSR	(Xu et al., 2013)
**11**	*Yr59*	*T. aestivum*	7BL	RGAP and SSR	(Zhou X. et al., 2014)
**12**	*Yr61*	*T. aestivum*	7AS	STS5467, STS5765b,	(Zhou X. et al., 2014)
**13**	*Yr64*	*T. durum*	1B	SSR	(Cheng et al., 2014)
**14**	*Yr65*	*T. durum*	1B	SSR	(Cheng et al., 2014)
**15**	*YrSD*	*T. aestivum*	5B	SSR	(Jing et al., 2013)
**16**	*YrHA*	*T. aestivum*	1AL	SSR	(Ma et al., 2013)
**17**	*YrSN104*	*T. aestivum*	1BS	SSR	(Asad et al., 2012)
**18**	*QYr.sicau*	Stripe rust of wheat	6B	SSR and KASP	(Hou S. et al., 2023)
**19**	*Yrpd.swust*	Stripe rust of wheat	7A	90K SNP	(Zhou et al., 2022)
**20**	*QYr.rcrrc*	Stripe rust of wheat	1B, 2A, 7D	SNP	(Tehseen et al., 2022)

## Mapped QTLs for smut diseases

Smut diseases are another important challenge faced by wheat crop. The pathogens causing smut diseases in wheat belong to the family *Ustilaginaceae* and order, *Ustilaginales*. Wheat is mainly infected by two smut diseases namely loose smut of wheat (*Ustilago tritici*) and flag smut disease (*Urocystis agropyri*) (Ram and Singh, 2004). However, loose smut is more serious than flag smut. Flag smut disease of wheat has been a serious issue leading to reduced yield. However, with the adoption of resistant cultivars and seed dressings, the prevalence has decreased (Toor and Bariana, 2012). A doubled haploid (DH) population was generated by crossing an Australian variety with high levels of tolerance against flag smut ‘Diamondbird’ with the susceptible Chinese landrace TH3929 (Murray and Brennan, 2009). Diamondbird identification of chromosomal position affecting flag smut resistance was recognized using a linkage map of 386 markers. Five quantitative trait loci (QTL) with significant impacts on flag smut resistance were discovered. The QTLs exposed by Diamondbird included QFs.sun-3AL, Qfs.sun6AS, Qcs.sun1BL, Qfsun5BS, and Qqs.sun3AS. Sequences of three or more QTLs were found in DH lines with low flag smut levels (Toor et al., 2013). In a high-density genetic map, the SNPs and 426 SSRs were mapped to 16 linkage groups spanning 3008.4 cM with an average intermarker gap of 0.2 cM. The blackbird variety of Nevski was crossed with the loose smut-sensitive durum (*Triticum turgidum* L.) Strongfield cultivar. This study has found three significant quantitative trait loci (QTL) to produce resistance against loose smut of wheat. The major QTL on 6B was stable to produce 74% phenotypic variation against all different races. The QTL QUt.spa-6B.2 could be useful to generate resistance against multiple strains of loose smut disease (Kumar et al., 2018). Six genes for loose smut resistance (Ut1-Ut6) were detected and reported based on the information provided by (Kassa et al., 2014). In the literature, from 34 genotypes of wheat, Krivchenko and Bakhareva identified 52 genes for resistance against loose smut; there were 11 recessive genes, and the remainder were dominant (Krivchenko and Bakhareva, 1984). Furthermore, Ut8, Ut9, and Ut10 on chromosomes 3A, 6B and 6D were also discovered (Knox et al., 2014). In the differential line TD-14, Ut11 was found and recognized as a strong resistance gene to loose smut disease. Thambugala et al. (2020) suggested that Ut11 showed resistance to *U. tritici* race T2, whereas races T9 and T39 did not. QUt.mrc-5B was responsible for the semi-resistant to smut phenotype and indicated resistance to all three races; however, it was less efficient against race T2. The genes and SNP markers linked with the QUt.mrc-5B, and Ut11 QTLs could be used in spring wheat breeding programs for marker-assisted selection. Many QTLs linked to smut disease resistance are given in **Table 3**.

**Table 3:** QTLs reported to be associated with smut disease resistance genes in wheat, their origin, chromosomal positioning and linked molecular markers.

Sr. No.	QTLs	Origin	Position	A. M. C	Source
**1**	*Stb1*	*T. aestivum*	5BL	qRT‐PCR, SSR	(Karlstedt, 2020)
**2**	*Stb18*	*T. aestivum*	6DS	SSR	(Tabib Ghaffary et al., 2011)
**3**	*StbSm3*	*T. aestivum*	3AS	–	(Brown et al., 2015)
**4**	*StbWW*	*T. aestivum*	1BS	–	(Raman et al., 2009)
**5**	*Stb6*	*T. aestivum*	3AS	–	(McCartney et al., 2003)
**6**	*Stb16q*	*T. aestivum*	3DL	–	(Ghaffary et al., 2012)
**7**	*Qsng.sfr.3BS*	*T. aestivum*	3BS	–	(Schnurbusch et al., 2003)
**8**	*Qsnb.fcu-1A*	*T. aestivum*	1A	–	(Abeysekara et al., 2009)
**9**	*Fhb1*	*T. aestivum*	3BS, 5AS	–	(Cuthbert et al., 2007)
**10**	*Fhb2*	*T. aestivum*	6BS	–	(Cuthbert et al., 2007)
**11**	*Fhb3*	*Leymus racemosus*	7AL	SSR	(Guo et al., 2015)
**12**	*Fhb4*	*T. aestivum*	4BL	SSR	(Guo et al., 2015)
**13**	*Fhb5*	*T. aestivum*	5AS	SSR	(Guo et al., 2015)
**14**	*Fhb6*	*Elymus tsukushiensis*	1AS	SSR	(Guo et al., 2015)
**15**	*Fhb7*	*Thinopyrum ponticum*	7DS	SSR	(Guo et al., 2015)

## Mapped QTLs for wheat nematodes

Plant parasitic nematodes (PPNs) are significant pathogens of wheat and cause various diseases with substantial economic losses (Ali et al., 2019). Several nematode species have been reported in wheat. For instance, cereal cyst nematodes (*Heterodera* spp.), root lesion nematodes (*Pratylenchus* spp.), root knot nematodes (*Meloidogyne* spp.), seed gall nematodes (*Anguina* spp.) and ring nematode (*Mesocriconema* spp.) (Seid et al., 2021). These parasitic nematodes use their stylet to enter the root cortex and release important enzymes in the form of secretions causing rupture of the cell wall (Ali et al., 2017). Plants are unable to adequately absorb water and nutrients from the soil due to the establishment of feeding sites on the vasculature of plant roots, resulting in water shortage and subsequent wilting. Nematodes of wheat cultivation have been recorded in Syria, Mexico, Canada, China, Yugoslavia, Morocco, Iran, India, Turkey, Pakistan, Algeria, Bangladesh, Australia, and the United States. Nematologists in cereal research prioritize enhancing nematode resistance in wheat for higher production. This generally concerned natural breeding selection of resistant germplasm and the discovery of QTLs linked to resistance genes. To improve resistance to *Heterodera avenae*, (Barloy et al., 2007) found that 9 resistance genes (e.g., Cre1 and Cre8 from *T. aestivum*; Cre3 and Cre4 from *Ae. tauschii* Coss.; Cre2, Cre5 and Cre6 from *Ae. ventricosa* (Zhuk.); Cre7 from *Ae. triuncialis* L.; CreR from rye and CreV from *Dasypium villosum* L.) were moved into common wheat from wild taxa such *Aegilops* and some other *Triticum* spp. However, *Rlnn1* is the only resistance gene identified in wheat that confers resistance to root knot lesion nematodes (Seid et al., 2021). The QTLs associated with CCN resistance are found on hexaploidy wheat chromosomes 1A, 1D, 4D, 5A, 5B, 5D, 6A, 6B, 7A, and 7D (Mulki et al., 2013). Likewise, Baloch et al. (2015) analyzed and provided complete data on the transfer of these genes and their related QTLs from wild relatives to common wheat. Consequently, MAS for cereal cyst nematode resistance in wheat is now widely utilized to discover resistant genotypes in Australia (Smiley et al., 2017). The Cre2 and Cre4 genes of *Aegilops* spp., as well as an unknown gene from the wheat line AUS4930, offered broad-spectrum resistance against multiple *Heterodera* species and pathotypes. CIMMYT coordinated the establishment of these loci in wheat in several parts of the globe, including Cre1 to Cre7, with a high level of resistance to CCNs. In addition, 11 DArT markers linked to CCN resistance have been identified, which can lead to the discovery of additional resistance loci and implements that could be valuable in wheat breeding programs (Dababat et al., 2016). In Australia, a combination of pot testing and MAS has been utilized to effectively decrease CCN infestation levels and losses (Ogbonnaya et al., 2009). Several studies suggest that using resistant cultivars can inhibit nematode reproduction and densities, while a wheat cultivar tolerant to *P. thornei* conserves development and production (Robinson et al., 2019). The GS50a bread wheat line is the first reported source of partial resistance against P*. thornei* produced from a highly infested wheat field (Thompson et al., 1999). In wheat, several QTLs have been discovered, and these resistance sources could be used for breeding attempts (Zwart et al., 2010). Furthermore, (Toktay et al., 2006) discovered resistance QTLs for *P. thornei* on chromosomes 1B, 2B, 3B, 4D, and 6D. Many QTLs linked to nematode disease resistance are given in **Table 4**.

**Table 4:** QTLs reported to be associated with plant parasitic nematodes resistance genes in wheat, their origin, chromosomal position and linked molecular markers.

Sr. No.	QTLs	Origin	Position	A. M. C	Source
**1**	*Cre1*	*T. aestivum*	2BL	microsatellite XGWM 301	Barloy et al., 2007
**2**	*Cre8*	*T. aestivum, Aegilops ventricosa*	6B	RFLP	(Williams et al., 2003)
**3**	*Cre3*	*Aegilops tauschii*	2DL	SSR (Xgwm301)	(Al-Doss et al., 2010)
**4**	*Cre4*	*Aegilops tauschii*	2DL		(Eastwood et al., 1991)
**5**	*Cre7*	*Aegilops triuncialis*	2BL		(Montes et al., 2008)
**6**	*Cre5*	*Aegilops ventricosa*	2AS	Microsatellite	(Williams et al., 2006)
**7**	*Cre6*	*Aegilops ventricosa*	5N		(Ogbonnaya et al., 2001)
	*Cre8*	*T. aestivum, Aegilops ventricosa*	6B	RFLP	(Williams et al., 2003)
**8**	*QRlnt.lrc*	*T. aestivum*	6DS	(AFLP)	(Zwart et al., 2005)
**9**	*QRlnn.lrc*	*T. aestivum*	6DS	(AFLP)	(Zwart et al., 2005)
**10**	*Cre2*	*Aegilops ventricosa*			(Delibes et al., 1993)
**11**	*QRlnt.sk-2B*	*T. aestivum*	2B	SSR and SNP	(Rahman et al., 2020)
**12**	*QRlnt.sk-6D*	*T. aestivum*	6D	SSR and SNP	(Rahman et al., 2020)
**13**	*QCre-ma7D*	*Triticum aestivum*	7DL	KASP (SNP)	(Cui et al., 2020)
**14**	*QCre-ma2A*	*T. aestivum*	2AS	KASP (SNP)	(Cui et al., 2020)
**15**	*QCcn-1B*	*F4 wheat population*	1BS	SSR (Xwmc85-1B)	(Al-Ateeq et al., 2021)
**16**	*TaPrx113-F*	*T. aestivum*		SSR and RAPD	(Al-Doss et al., 2010)
**17**	*TaPrx112-D*	*T. aestivum*	2B	SSR and RAPD	(Al-Doss et al., 2010)
**18**	*CreY*	*T. aestivum*	3BL	Microsatellite (OPY16)	(Dababat et al., 2016)
**19**	*Rlnn1*	*T. aestivum*	7AL	AFLP (Xcdo347-7A)	(Williams et al., 2002)
**20**	*CreX*	*Variabilis L.*			(Cui et al., 2020)
**21**	*CreR*	*Secale cereale L.*			(Cui et al., 2020)
**22**	*CreV*	*Dasypyrum villosum L.*	6VL	STS	(Zhang et al., 2016)
**23**	*CreZ*	*T. aestivum*	EU327996	Real time PCR	(Dababat et al., 2016)

## Mapped QTLs for tan spot of wheat

Tan spot of wheat is a fungal disease caused by *Pyrenophora tritici-repentis*, which affects wheat plants and can lead to significant yield losses if left untreated. The disease is common in wheat-growing regions worldwide and can be identified by small, dark, oval-shaped lesions with tan centers on the lower leaves of the plant, which can eventually lead to large necrotic spots and yellowing of the leaves (Muqaddasi et al., 2021). The disease cycle of tan spot involves the overwintering of the pathogen in crop debris and its survival in soil for several years. In the spring, spores are produced, and infection can occur at any growth stage, favored by warm and humid conditions (Istifadah and McGee, 2006). Effective management of tan spot in wheat involves the integration of disease management practices but the most effective way to manage tan spot disease is to plant resistant wheat cultivars. The identification of quantitative trait loci (QTLs) associated with tan spot resistance has facilitated the development of new resistant wheat cultivars. Research has identified several QTLs associated with tan spot resistance in wheat, including chromosomes 1B, 2B, 2D, 3A, 3B, 4A, 4B, 4D, 5A, 5B, 6A, 6B, and 7D. However, the degree of resistance conferred by these QTLs varies, and breeders must carefully select which QTLs to incorporate into their breeding programs to achieve the desired level of resistance (Phuke et al., 2020). In the study, a group of wheat recombinant inbred lines were examined for their response to various tan spot disease isolates, with the lines originating from resistant and susceptible varieties. The Tsn1 locus was found to be significantly linked to the disease caused by ToxA-producing isolates, although the extent of the association differed among the isolates. Another locus on chromosome arm 7DS was identified as being specifically related to an isolate that did not produce ToxA. In addition, other QTL on 5DL and 7BS were discovered to be race-nonspecific and linked to tan spot caused by multiple isolates. These findings suggest that the wheat-tan spot patho system is more intricate than previously thought, and that race-nonspecific resistance QTL plays an important role in controlling the response to tan spot (Faris et al., 2012). Further, the study was conducted to assess the reaction of a population of recombinant inbred lines derived from a cross between Grandin and BR34 wheat varieties to different races of the tan spot pathogen. The study identified QTLs on chromosomes 1B and 3B that were significantly associated with resistance to all four races, with varying effects for each race. The 1B QTL explained 13% to 29% of the variation, and the 3B QTL explained 13% to 41%. Although minor QTLs were detected, they were not linked to resistance to all races. Surprisingly, the production of Ptr ToxA by races 1 and 2 did not contribute significantly to disease development. The race-nonspecific resistance derived from BR34 may be more important than the gene-for-gene interaction typically associated with the wheat-Ptr system (Faris et al., 2012).

## Genome wide association studies for disease-resistance in wheat

Although bi-parental QTL mapping has been successful, it usually takes years to develop a mapping population, and gene discovery is limited to the genetic background of only two parents (Cockram and Mackay, 2018). The more recent techniques used to understand natural variations and associate a particular character to a particular genotype are genome-wide association studies (GWAS), also known as genome-wide association mapping (Baloch et al., 2015). The latest progress and accessibility of genotyping by sequencing (GBS) techniques in wheat, which was used to characterize a wide array of hexaploid cultivars from many parts of the world, is facilitating the use of SNPs in GWAS (Muhammad et al., 2020). Different technologies have been used for the identification of SNPs after sequencing approaches. For instance, SNP arrays, such as 90K arrays, have been used in genome-wide association studies to identify genomic regions and/or markers for several traits, such as grain asparagine contents, resistance to Hessian flies, disease resistance, grain yield and frost tolerance in wheat (Muhu-Din Ahmed et al., 2022).

GWAS is a useful technique to identify significant markers for characteristics in wheat (Verges et al., 2021). GWASs have been reported against the most threatening fungal diseases to wheat production, including rust, smut, and bunt diseases (Goutam et al., 2015). Leaf rust is the most common among all wheat rust which infect most wheat growing fields (Vanzetti et al., 2011). Leaf resistance (*Lr*) genes have been reported to be linked with a wide range of markers (Imbaby et al., 2014). GWAS also reported in fusarium head blight (FHB) (Verges et al., 2021) resistance in winter wheat lines, suggesting that GWAS is the most effective approach for FHB resistance (Savadi et al., 2018). Furthermore, there are sixteen race-specific resistance genes to common bunts from Bt1 to Bt15 that have been reported (Goates, 2012). Furthermore, 123 SNPs were shown to be linked with resistance on fourteen chromosomes in a genome-wide association study (Mourad et al., 2018). This GWAS and molecular markers could be useful tools for the characterization of DB resistance in bread wheat. A QTL that had been previously identified on chromosome 6DS, as well as a newly discovered locus on the same chromosome, were shown to explain 9-15% of phenotypic variation (Gordon et al., 2020).

**Figure 3:**
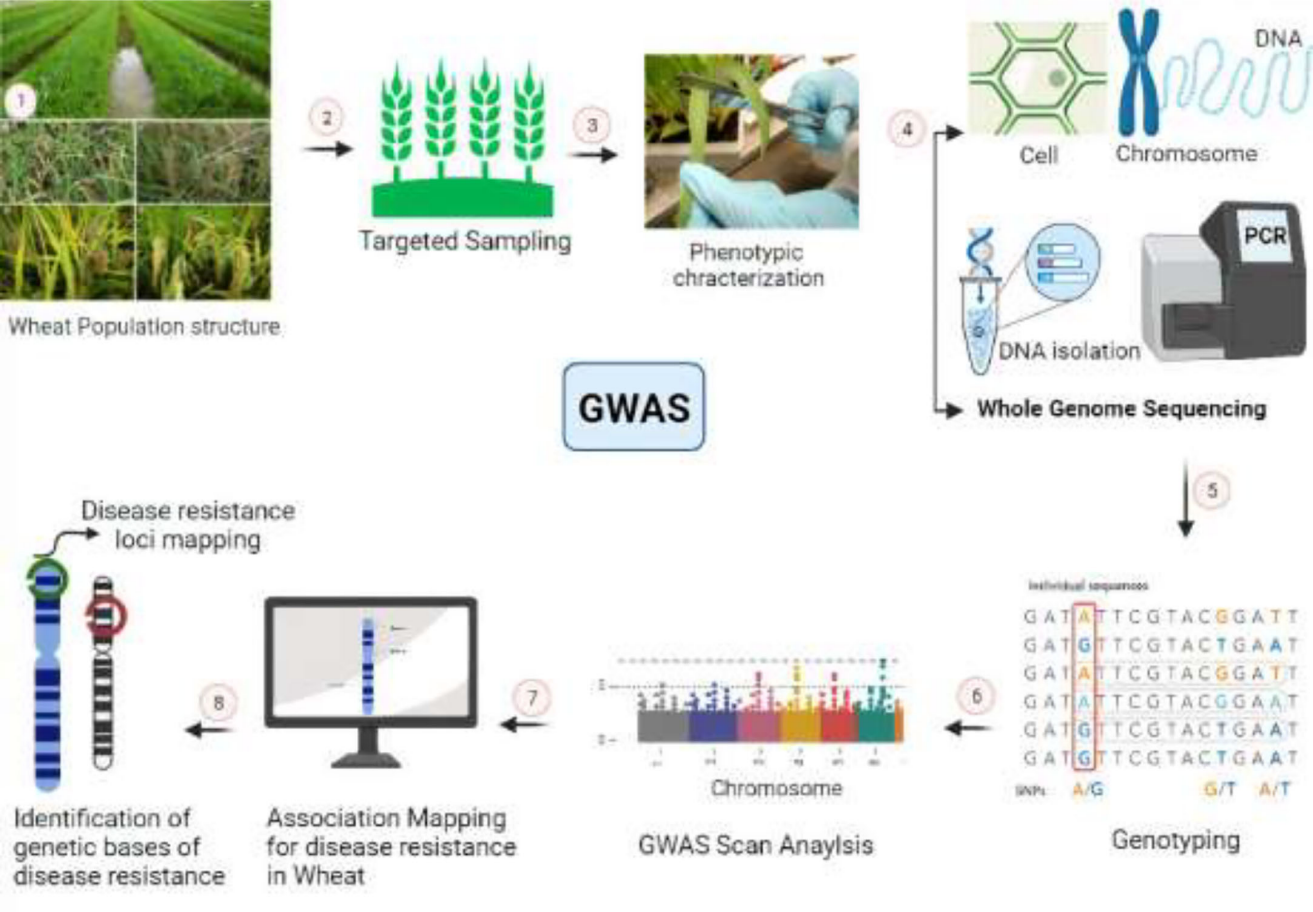
Genome-wide association studies demonstrating work scheme for the development of disease resistance in wheat. 1, 2: Cultivation of wheat for large scale field trials and pathogen application. 3: Phenotypic evaluation and measurement of disease resistance in wheat-pathogen reactions. 4: Whole-genome sequencing, assembly, and annotation of selected lines. 5: Alignment of reads with reference genome and identification of molecular markers i.e., SNPs. 6: Statistical data analysis and predictions of associated markers with QTLs of interest. 7, 8: Chromosomal mapping of identified QTLs and their further functional validation.

GWAS could be a potential technique for identifying marker trait relationships for biotic and abiotic resistance (Ali et al., 2019). There have not been many GWAS reports in wheat of resistance to nematodes (Würschum et al., 2012), as only one gene, *Rlnn1*, came from an Australian spring wheat cultivar (Williams et al., 2002). Additionally, (Dababat et al., 2016) discovered nine significant marker trait associations (MTAs) on chromosomes 1D, 2A, 3B, 5B, and 7A related to *P. thornei* resistance using GWAS. Genome-wide association analyses have been routinely used to link nematode resistance or susceptibility to specific genomic areas. It is commonly known that different resistance sources against different diseases in common wheat are derived from wild wheat families by breeding strategies. These advancements were made possible by the crop plants’ genetic variety (Ogbonnaya et al., 2001). Furthermore, R genes encode surface immune receptor-like kinases (RLKs), and intracellular immune receptors include nucleotide-binding leucine-rich repeat proteins (NLRs) that can identify toxic molecules or avirulence (*Avr*) proteins in pathogens directly or indirectly (Monteiro and Nishimura, 2018). Most resistance genes (*R*- genes) are linked to race-specific interactions, also known as “gene for gene” interactions (Flor, 1971), which are easily broken down within the field, as pathogens can change to avoid host identification by modifying the homologous avirulence gene. The use of high-density single nucleotide polymorphism (SNP) maps in genome-wide association studies (GWAS) has proven successful in discovering many important marker traits linked to Fusarium head blight (FHB) traits (Khan et al., 2020). Different important steps of GWAS for disease resistance in wheat are shown in **Figure 3**.

## CRISPR/Cas-9 mechanism- a breakthrough genome editing tool for enhancing wheat resistance to diseases

**Figure 4:**
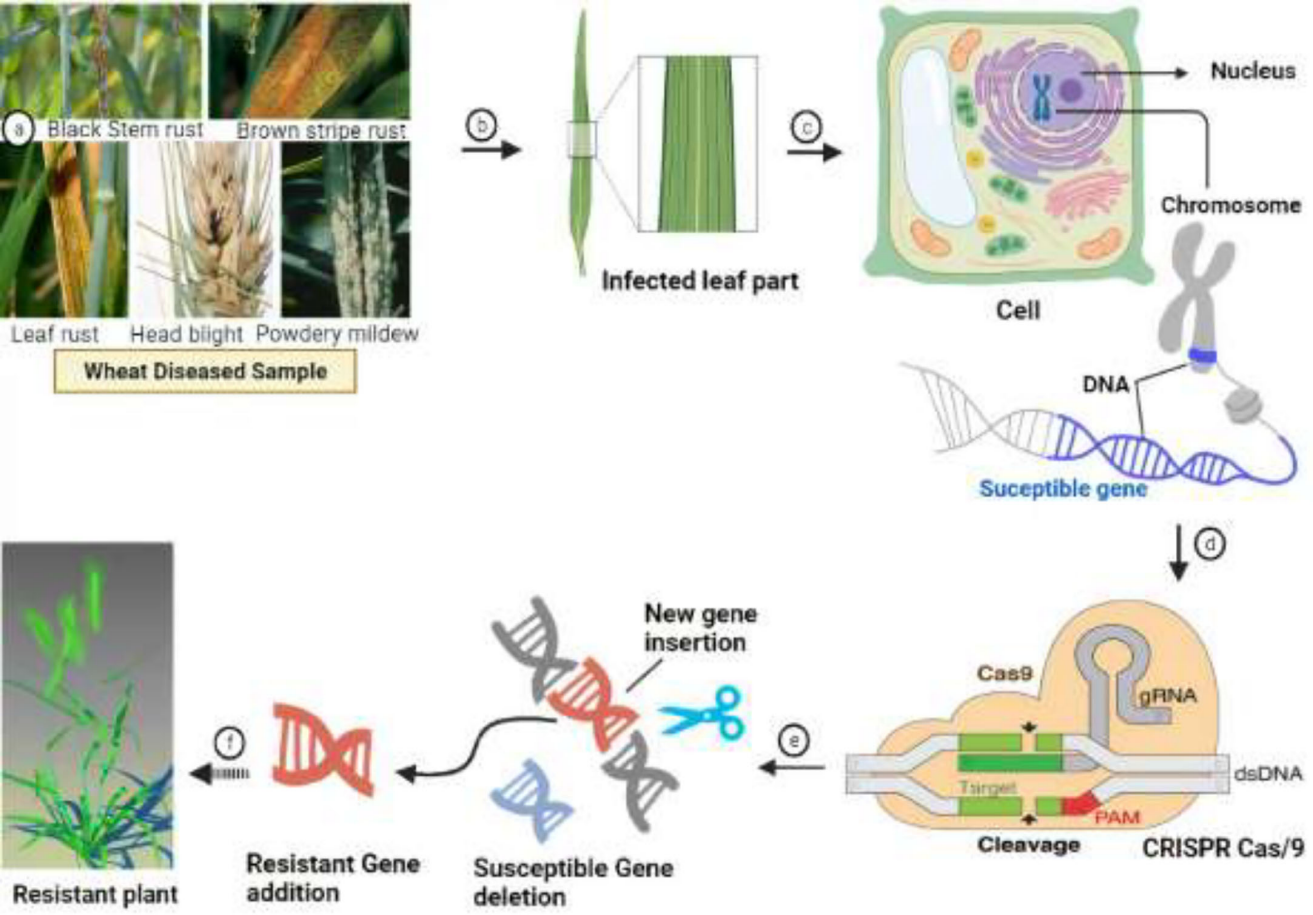
Overview of the CRISPR-Cas9 genome editing system and figurative description of how it works for the development of disease resistance in wheat. **(A, B)** Identification and assessment of wheat germplasm susceptible to pathogens. **(C)** In-depth molecular analysis of the wheat genome *via* various genomics-transcriptomics techniques and identification of susceptible/Avr genes that favor pathogen growth on wheat plants. **(D)** Description of the molecular mechanism depicting how the CRISPR-Cas9 construct identifies the Avr genes scanning the wheat genome. **(E)** Deletion or replacement of Avr genes with resistant R genes that hinder pathogen colonization. **(F)** Development of healthy and resistant wheat plants for future cultivation.

**Figure 5:**
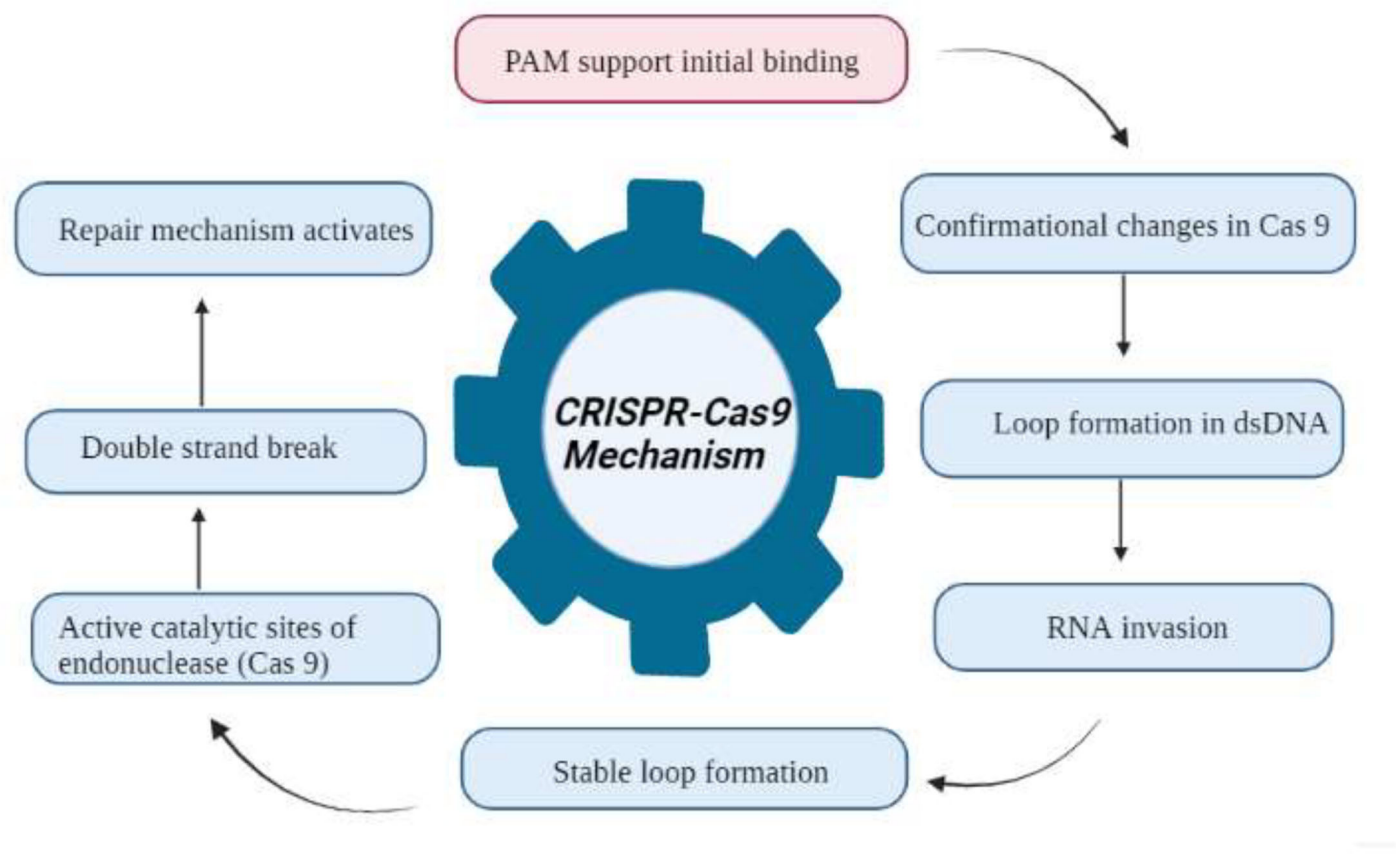
Flowchart of the CRISPR Cas9 mechanism for the development of disease resistance in wheat. The recognition, cleavage, and repair phases are the main stages of the CRISPR/Cas-9-based genome editing system. The PAM sequence is present on the noncomplementary strand, and it is on the strand of DNA that contains the same DNA sequence as the target crRNA. After initial recognition at the target locus, CRISPR/Cas9 causes precise double-strand breaks in the target DNA, which is further followed by DNA repair processes.

Wheat hexaploidy (2n = 6x = 42, AABBDD genome) and gene functions make it difficult to choose a suitable phenotype through genetic advances (Adamski et al., 2020). To achieve worldwide food and environmental security, genomic approaches must be used to improve wheat yield and resistance (Li J. et al., 2021). Genome-editing techniques have transformed plant studies and have a great capacity for crop enhancement. In the agriculture sector, disease-resistant varieties in breeding programs are the most cost-effective and environmentally friendly approach to disease management (**Figure 4**) (Choudhary et al., 2022). As a result, increasing crop breeding policies that promote disease resistance require an awareness of plant–pathogen interactions and crop immune mechanisms (Li J. et al., 2021). Among genome editing tools, CRISPR/Cas9 is a promising gene editing technology in crop plants because it is specialized, highly specific, a multigene editor, and highly useful (Rao and Wang, 2021). Compared to other gene editing methods, such as transcription activator-like effector nucleases (TALENS) (Sun and Zhao, 2013) and zinc finger nucleases (ZFNs) (Townsend et al., 2009), CRISPR/Cas9 is an RNA-guided apparatus enabling efficient and accurate genome editing. CRISPR (clustered regularly interspaced palindromic repeats) Cas9 (CRISPR-associated protein) is a new wave of genome editing that is quick, easy, and cheap. For a long time, it has been utilized as a bacterial immune system and has recently made a significant advancement in the field of genome editing (**Figure 5**) (Yang et al., 2022). In 2012, CRISPR Cas9 was first described for genome editing by American scientist Doudna and French microbiologist Emmanuelle Charpentier (Cohen, 2017). In addition to wheat, many other crop species have benefited from the CRISPR/Cas9-based genome editing system. For example, this system is employed in more than 20 crop species to improve production, biotic and abiotic stress management, and other features (Farooq et al., 2021). As in rice, CRISPR-mediated targeting of the OsERF922 gene has led to resistance against bacterial blight and rice blast (Romero and Gatica-Arias, 2019). The ARGOS8 gene in maize has been mutated to improve grain yield during drought stress (Chilcoat et al., 2017). The *ALS1* gene in potatoes was also targeted for resistance to herbicides (Danilo et al., 2019). Because CRISPR/Cas9 is a sequence specific nuclease, it could be used to influence plant defensive mechanisms against invading pathogens (Tyagi et al., 2021).

Wheat production and disease resistance have both benefited from the adoption of CRISPR/Cas9. The use of CRISPR/Cas9 technology has significantly impacted the production of wheat and improved its resistance to diseases. Additionally, CRISPR/Cas9 can be used to introduce new traits into wheat, such as improved grain quality, higher yield potential, or tolerance to abiotic stress. By combining these traits with disease resistance, the overall productivity of the crop can be increased (Zhang et al., 2021). For instance, lipoxygenase (LOX) has various roles in plant growth and development and resistance against diseases. *TaLOX2* knockout improves wheat shelf life by altering grain size and weight (Shan et al., 2014). The RING-type E3 ligase-encoding gene *TaGW2* was knocked out, resulting in longer and wider wheat grains and higher grain yields (Zhang et al., 2018). Multiplexed gene editing (MGE) based on CRISPR-Cas9 is an effective is an effective tool for modifying many genomic zones at the same time to control diverse agronomic traits. The MGE created by combining tandemly arrayed tRNA–gRNA units resulted in genetic mutations in the wheat genes *TaGW2, TaLpx-1, and TaMLO* (Wang et al., 2018). The desired mutation and resistance in wheat plants altered by CRISPR/Cas9 in one of the three MLO homoalleles (*TaMLO-A1*) demonstrated increased resistance against *Blumeria graminis* f. sp. *tritici* infection. This finding explains the importance of *TaMLO* genes against powdery mildew disease once again (Wang et al., 2014). Likewise, resistance is improved to powdery mildew disease (*Blumeria graminis* f. sp) of wheat TaEDR1/cds area CRISPR/Cas9 (Zhang et al., 2017). Wheat dwarf virus (WDV) is an economically significant virus that is spread by insects (Kis et al., 2019). In model plants, direct use of CRISPR/Cas9 technology against *geminivirus* replication has been explained (Zaidi et al., 2016). WDV Guide4-Guard Lines 1, 3, and 4 showed no signs of viral infection, and neither northern blot nor PCR testing revealed the virus’s existence. These findings suggested that these lines are completely resistant to WDV infection (Kis et al., 2019). Similarly, in the free-living nematode *Caenorhabditis elegans*, genome editing utilizing the CRISPR/Cas9 system has newly been recognized (Paix et al., 2017). This breakthrough would lead to the identification of numerous key genes participating in several nematode physiological systems (Ullah et al., 2020). However, the CRISPR/Cas9 process can be complex and time-consuming, especially when multiple genes are targeted. There is also a risk of off-target effects, where the CRISPR/Cas9 system may edit unintended regions of the genome.

## Reprogramming of wheat genome through gene pyramiding/stacking using molecular markers

Gene pyramiding is an important breeding strategy in wheat breeding programs that involves the integration of multiple resistance genes into a single cultivar to enhance resistance against biotic and abiotic diseases. This strategy is particularly useful in the development of cultivars that can withstand multiple stresses, thus providing farmers with more resilient and productive crops (Dormatey et al., 2020). One suitable way to achieve functional gene pyramiding is using molecular markers. Markers are DNA sequences that can be used to identify genes associated with specific traits or to track the inheritance of these genes during breeding programs. By using markers, breeders can identify plants that have multiple resistance genes and select them for further breeding (Ye and Smith, 2008). Many global and key national wheat breeding programs have adopted functional gene stacking strategy using markers to develop wheat cultivars with improved resistance to multiple stresses. For example, in India, the Indian Council of Agricultural Research (ICAR) has developed wheat cultivars with resistance to multiple diseases, including rusts and powdery mildew, using functional gene pyramiding. These cultivars have been widely adopted by farmers and have contributed to increased wheat productivity and food security in the country (Kumaran et al., 2021). Similarly, the Chinese Academy of Agricultural Sciences (CAAS) has also developed wheat cultivars with multiple disease resistances using functional gene pyramiding. These cultivars have been widely adopted by farmers in China and have contributed to increased wheat yields and improved food security (Li et al., 2014). Likewise, gene pyramiding has been used to develop cereal cyst nematode resistance with multiple genes in wheat. This involved combining resistance genes from various origins, such as Cre1 located on the 2BL chromosome arm, Cre3 found on the 2DL chromosome arm, and Cre8 situated on the 6BL chromosome arm. Specific molecular markers linked through PCR were then utilized to track each gene individually (Barloy et al., 2007; Ogbonnaya et al., 2009). Overall, functional gene pyramid using markers is an effective breeding strategy for developing wheat cultivars with improved disease resistance. It has been successfully adopted by many global and key national wheat breeding programs, and it is likely to continue to play an important role in the development of resilient and productive wheat cultivars in the future.

## Concluding remarks and perspectives

The objective of this review was to provide an overview of reported whole effective QTLs and an understanding of numerous modern molecular techniques that could be used to improve pathogen resistance in bread wheat. It is concluded that the best strategy to manage major wheat diseases is to develop modern genetic resistance, which can be achieved by identifying genes/quantitative trait loci (QTLs) and molecular markers. Overall, innovative genetic methods based on molecular marker tools are a recommended viable option for improving resistant cultivars against diseases. Wheat genomics has been revolutionized by the advancement of molecular markers such as SSLPs, RFLPs, SSRs, ISSR, AFLPs, SCAR, SNPs, and DArT within the previous two decades. Marker-assisted selection and functional genomics techniques are both efficient approaches for developing resistant germplasm. Among them, QTL mapping is the most effective approach to determine the exact position of a gene. Furthermore, one of the most widely used transgenic methods for silencing the gene expression of pathogen effector-encoding genes is RNA interference (RNAi) to improve disease resistance and different traits in crop plants (Majumdar et al., 2017). By introducing specific dsRNA sequences to confer resistance in host plants, RNAi could be used to downregulate pathogen genes involved in invasion, growth, and pathogenicity in plants. This approach is mostly used as host-induced gene silencing (HIGS) to suppress wheat pathogens, i.e., nematodes. In recent years, resistance engineering has focused on identifying and silencing genes that contribute to facilitating pathogen attack, as these factors may provide more persistent resistance than R genes (Schaefer et al., 2020). Furthermore, the nucleotide-binding site and leucine-rich repeat (NBS-LRR or NLR) proteins are encoded by the largest family of R genes (Gururani et al., 2012). Most R genes, as well as linked components, aid pathogen detection and provide resistance to a subset of pathogens, or races, that produce certain effector proteins gene-for-gene. The discovery, conservation, and effective transfer of R genes between plant species has resulted in the development of long-lasting resistance and crop protection against a variety of infections. Engineered R genes for conferring resistance to a broad variety of pathogens (Jiang et al., 2020) could be a potential method to improve disease resistance in wheat. Molecular markers provide several advantages over traditional phenotype-based alternatives, including that they are stable and detectable in all defensive statuses. A microarray-based approach throughput high molecular marker has been established for evaluating thousands of DNA samples using a co-dominant molecular marker on a glass slide.

They are valuable tools not only for whole-genome transcript profiling but also for the identification of genotypes and polymorphisms. The microarrays used in DNA-chip technology are microscopic arrays of nucleic acid molecules immobilized on a solid surface, typically glass, for biochemical examination. DNA technologies are favorable and powerful means for identifying taxa at different taxonomic levels, including species, subspecies, variety, and strain, because they produce consistent results regardless of tissue origin, physiological conditions, environmental factors, sample harvest, storage, and processing. With the growing demand for high-quality herbs, the requirement for DNA verification to ensure plant effectiveness will increase. The European Union (EU) has created microarrays to speed up the discovery of DNA polymorphisms in plants. According to the EU, the technique could be used to diagnose plant diseases as well as improve crop breeding. The risk of reduced genetic diversity in the wheat crop if only a small number of resistance genes are used in breeding programs.

## Future scenarios and recommendations

Regarding the future recommendations for the above data analysis, several additional potentially helpful study directions for proper identification and early detection of wheat pathogens are important to prevent the spread of the fungus that causes major diseases in wheat. The development of disease-resistant cultivars is insufficient to save plants and feed a rising population, particularly in developing nations. Major diseases of wheat result in significant production losses, forcing researchers to generate multiple pathogen-resistant varieties of wheat. Multiple disease resistance is a vital tool against pathogens in sustainable agriculture, it is cost-effective for both farmers and the environment. Similarly, remote sensing technology could increase the depth and dependability of disease surveys conducted annually. Future tasks include acquiring more suitable markers to enhance wheat breeders’ strengths. In the literature, integrating molecular breeding works among a few national program partners is also seen as an important issue. One of the main priorities of wheat researchers is making wheat globally competitive by lowering cultivation costs and enhancing farmer profitability. On the other hand, genomic transformation will continue to be a vital tool for studying gene functions and evaluating the value of new sequences. In addition, the peptide method keeps only one functional domain to interfere with the target functions. Additionally, the mechanism of pathogen-derived resistance known as posttranscriptional gene silencing (PTGS) has been discovered. PTGS-based resistance traits are typically strong. The technique represents a potential novel concept that could be utilized for yeast two-hybrid systems to select target-specific peptides (“aptamers”) to modify block protein functions *in vivo*. Additionally, in the early days, traditional plant breeding techniques were used in the field of agriculture. However, some drawbacks are that they cannot transfer specific traits to species. Hence, new agricultural enhancement approaches can transfer genes between different species, including vegetatively reproducing plants. It is critical to uncover novel resistance genes/gene families to attain the goal of obtaining disease-resistant plants (Vega-Arreguín et al., 2017). For the identification of novel genes, advanced approaches such as virus-induced gene silencing (VIGS), host-induced gene silencing (HIGS), and mutant screening have been widely used (Sawyer and Labbé, 2021). Host-induced gene silencing (HIGS) is a transgene-based strategy with highly specific siRNA design, off-target effects, and productive technology. By altering gene expression in a precise way, these approaches can provide plant protection against a wide range of plant pathogens. Additionally, spray-induced gene silencing (SIGS) is a nontransgenic technique that uses a spray of tiny RNA molecules to regulate the target gene(s) in a highly targeted manner (Taning et al., 2020). Moreover, sequence-specific nucleases (SSNs) could be utilized to create a broad spectrum of sequence alterations for disease resistance in plant genes. Moreover, the most effective way to reduce the impact of plant diseases is to contain the pathogens at their birthplace. New plant biotechnologies can detect a wide range of uncharacterized pathogens, posing a major task for quarantine standards. The laws of plant quarantine can be administered both at the national level and at the regional level within a country. The molecular breeding of highly wanted wheat cultivars will undoubtedly benefit from the incorporation of developing novel biotechnologies.

## Author contributions

MJ wrote the manuscript and worked on figures. MA and AZ edited and reviewed the manuscript. GM-U-D worked on the tables and revised the manuscript. LGfounded,supervised, edited, reviewed and approved the final manuscript. All authors contributed to the article and approved the submitted version.

## References

[B1] Abeysekara N. S. Friesen T. L. Keller B. Faris J. D. (2009). Identification and characterization of a novel host-toxin interaction in the wheat *Stagonospora nodorum* pathosystem. Theor. Appl. Genet. 120, 117–126. doi: 10.1007/S00122-009-1163-6/FIGURES/4 19816671

[B2] Adamski N. Borrill P. Brinton J. Harrington S. (2020). A roadmap for gene functional characterization in crops with large genomes. Elife; 9, e55646. doi: 10.7554/eLife.55646 32208137 PMC7093151

[B3] Ahmad S. Wei X. Sheng Z. Hu P. Tang S. (2020). CRISPR/Cas9 for development of disease resistance in plants: recent progress, limitations and future prospects. Brief. Funct. Genom. 19, 26–39. doi: 10.1093/bfgp/elz041 31915817

[B4] Akın B. Yüce S. Singh R. Braun H. J. Zencirci N. Morgunov A.. (2013). Leaf rust (Puccinia triticina) resistance genes determination using race differentials and molecular markers in winter–facultative wheat (*Triticum aestivum L*.). Agric. Res. J. 3 (6), 167–177. Available at: https://www.researchgate.net/publication/270574221.

[B5] Al-Ateeq T. K. Al-Doss A. A. Al-Hazmi A. S. Ghazy A. I. Dawabah A. M. Motawei M. I. (2021). Molecular mapping of a novel QTL for resistance to cereal cyst nematode in F4 wheat population. Cereal Res. Commun. 50, 11–17. doi: 10.1007/s42976-021-00159-9

[B6] Al-Doss A. A. Al-Hazmi A. S. Dawabah A. A. Abdel-Mawgood A. A. Al-Rehiayani S. M. Al-Otayk S.. (2010). Impact of’Cre’and peroxidase genes of selected new wheat lines on cereal cyst nematode (‘*Heterodera Avenae*’Woll) resistance. Aust. J. Crop Sci. 4 (9), 737–743. doi: 10.3316/informit.859558578260383

[B7] Ali M. A. Azeem F. Abbas A. Joyia F. A. Li H. Dababat A. A. (2017). Transgenic strategies for enhancement of nematode resistance in plants. Front. Plant Sci. 8, 750. doi: 10.3389/fpls.2017.00750 28536595 PMC5422515

[B8] Ali M. A. Shahzadi M. Zahoor A. Dababat A. A. Toktay H. Bakhsh A.. (2019). Resistance to cereal cyst nematodes in wheat and barley: an emphasis on classical and modern approaches. Int. J. Mol. Sci. 20, 432. doi: 10.3390/ijms20020432 30669499 PMC6359373

[B9] Al-Maaroof E. M. Ali R. M. Mahmood H. A. Aziz T. M. (2016). Searching for resistance sources to wheat common bunt disease and efficiency of *Bt* genes against *Tilletia tritici* and *T. laevis* populations. Agric.& Forest. 61, 175–186. doi: 10.17707/AgricultForest.62.1.20

[B10] Amom T. Nongdam P. (2017). The use of molecular marker methods in plants: a review. Int. J. Curr. Res. Rev. 9, 1–7. doi: 10.7324/IJCRR.2017.9171

[B11] Aravindh R. Sivasamy M. Ganesamurthy K. Jayaprakash P. Gopalakrishnan C. Geetha M.. (2020). Marker assisted stacking/pyramiding of stem rust, leaf rust and powdery mildew disease resistance genes (Sr2/Lr27/Yr30, Sr24/Lr24 and Sr36/Pm6) for durable resistance in wheat (*Triticum aestivum* l.). Electron. J. Plant Breed. 11, 907–915. Available at: https://www.ejplantbreeding.org/index.php/EJPB/article/view/3643.

[B12] Asad M. A. Xia X. Wang C. He Z. (2012). Molecular mapping of stripe rust resistance gene YrSN104 in Chinese wheat line shaannong 104. Hereditas 149, 146–152. doi: 10.1111/j.1601-5223.2012.02261.x 22967144

[B13] Autrique E. Sorrells M. E. Singh R. Singh R. P. Tanksley S. D. Sorrells M. E. (1995). Molecular markers for four leaf rust resistance genes introgressed into wheat from wild relatives. Genome 38, 75–83. doi: 10.1139/g95-009 18470154

[B14] Baloch F. S. Cömertbay G. Özkan H. (2015). DNA Molecular markers for disease resistance in plant breeding with example in wheat. Nematodes Small Grain Cereals 159–166.

[B15] Baltes N. J. Hummel A. W. Konecna E. Cegan R. Bruns A. N. Bisaro D. M.. (2015). Conferring resistance to geminiviruses with the CRISPR–cas prokaryotic immune system. Nat. Plants 1, 1–4. doi: 10.1038/nplants.2015.145 PMC8612103 34824864

[B16] Bansal U. Bariana H. Wong D. Randhawa M. Wicker T. Hayden M.. (2014). Molecular mapping of an adult plant stem rust resistance gene Sr56 in winter wheat cultivar arina. Theor. Appl. Genet. 127, 1441–1448. doi: 10.1007/s00122-014-2311-1 24794977

[B17] Bansal U. K. Forrest K. L. Hayden M. J. Miah H. Singh D. Bariana H. S. (2011). Characterisation of a new stripe rust resistance gene Yr47 and its genetic association with the leaf rust resistance gene Lr52. Theor. Appl. Genet. 122, 1461–1466. doi: 10.1007/s00122-011-1545-4 21344185

[B18] Bansal U. K. Hayden M. J. Venkata B. P. Khanna R. Saini R. G. Bariana H. S. (2008). Genetic mapping of adult plant leaf rust resistance genes Lr48 and Lr49 in common wheat. Theor. Appl. Genet. 117, 307–312. doi: 10.1007/S00122-008-0775-6 18542911

[B19] Barloy D. Lemoine J. Abelard P. Tanguy A. M. Rivoal R. Jahier J. (2007). Marker-assisted pyramiding of two cereal cyst nematode resistance genes from aegilops variabilis in wheat. Mol. Breed. 20, 31–40. doi: 10.1007/S11032-006-9070-X

[B20] Begum H. Spindel J. E. Lalusin A. Borromeo T. Gregorio G. Hernandez J.. (2015). Genome-wide association mapping for yield and other agronomic traits in an elite breeding population of tropical rice (*Oryza sativa*). PloS One 10, e0119873. doi: 10.1371/journal.pone.0119873 25785447 PMC4364887

[B21] Bhanu A. N. Singh M. N. Srivastava K. Hemantaranjan A. (2016). Molecular mapping and breeding of physiological traits. Adv. Plants Agric. Res. 3, 10–15406. doi: 10.15406/apar.2016.03.00120

[B22] Bhardwaj S. C. Prashar M. Prasad P. (2014). “Ug99-future challenges,” in Future challenges in crop protection against fungal pathogens. fungal biology. Eds. Goyal A. Manoharachary C. (New York, NY: Springer). doi: 10.1007/978-1-4939-1188-2_8

[B23] Briggs F. N. (1933). A third genetic factor for resistance to bunt, *Tilletia tritici*, in wheat hybrids. J. Genet. 27, 435–441. doi: 10.1007/BF02981755 PMC1208447 17246711

[B24] Brown J. K. M. Chartrain L. Lasserre-Zuber P. Saintenac C. (2015). Genetics of resistance to *Zymoseptoria tritici* and applications to wheat breeding. Fungal Genet. Biol. 79, 33–41. doi: 10.1016/j.fgb.2015.04.017 26092788 PMC4510316

[B25] Brown-Guedira G. L. Singh S. Fritz A. K. (2003). Performance and mapping of leaf rust resistance transferred to wheat from *Triticum timopheevii* subsp. *armeniacum* . Phytopathology 93, 784–789. doi: 10.1094/PHYTO.2003.93.7.784 18943158

[B26] Bulos M. Echarte M. Sala C. (2006). Occurrence of the rust resistance gene Lr37 from *Aegilops ventricosa* in Argentine cultivars of wheat. Electron. J. Biotechnol. 9 (5), 1–15. doi: 10.2225/vol9-issue5-fulltext-14

[B27] Castlebury L. A. Carris L. M. Vánky K. (2005). Phylogenetic analysis of *Tilletia* and allied genera in order *Tilletiales* (*Ustilaginomycetes*; *Exobasidiomycetidae*) based on large subunit nuclear rDNA sequences. Mycologia 97, 888–900. doi: 10.1080/15572536.2006.11832780 16457358

[B28] Ceresini P. C. Castroagudín V. L. Rodrigues F. Á. Rios J. A. Eduardo Aucique-Pérez C. Moreira S. I.. (2018). Wheat blast: past, present, and future. Annu. Rev. Phytopathol. 56, 427–456. doi: 10.1146/annurev-phyto-080417-050036 29975608

[B29] Chen X. M. (2005). Epidemiology and control of stripe rust [*Puccinia striiformis* f. sp. tritici] on wheat. Can. J. Plant Pathol. 27, 314–337. doi: 10.1080/07060660509507230

[B30] Chen J. Guttieri M. J. Zhang J. Hole D. Souza E. Goates B. (2016). A novel QTL associated with dwarf bunt resistance in Idaho 444 winter wheat. Theor. Appl. Genet. 129, 2313–2322. doi: 10.1007/S00122-016-2783-2 27681089 PMC5121181

[B31] Cheng P. Xu L. S. Wang M. N. See D. R. Chen X. M. (2014). Molecular mapping of genes Yr64 and Yr65 for stripe rust resistance in hexaploid derivatives of durum wheat accessions PI 331260 and PI 480016. Theor. Appl. Genet. 127, 2267–2277. doi: 10.1007/s00122-014-2378-8 25142874

[B32] Chilcoat D. Liu Z.-B. Sander J. (2017). Use of CRISPR/Cas9 for crop improvement in maize and soybean. Prog. Mol. Biol. Transl. Sci. 149, 27–46. doi: 10.1016/bs.pmbts.2017.04.005 28712499

[B33] Choudhary A. Kumar A. Kaur H. Pandey V. Singh B. Mehta S. (2022). “Breeding strategies for developing disease-resistant wheat: present, past, and future,” in Cereal diseases: nanobiotechnological approaches for diagnosis and management (Singapore: Springer Nature Singapore), 137–161.

[B34] Ciechanowska I. Semagn K. McCallum B. Randhawa H. Strenzke K. Dhariwal R.. (2022). Quantitative trait locus mapping of rust resistance and agronomic traits in spring wheat. Can. J. Plant Sci. 102 (6), 1139–1150. doi: 10.1139/cjps-2022-0023

[B35] Ciucă M. (2011). A preliminary report on the identification of SSR markers for bunt (*Tilletia* sp.) resistance in wheat. Czech J. Genet. 47, S142–S145.

[B36] Cockram J. Mackay I. (2018). “Genetic mapping populations for conducting high-resolution trait mapping in plants,” in Plant genetics and molecular biology. advances in biochemical Engineering/Biotechnology, vol. vol 164 . Eds. Varshney R. Pandey M. Chitikineni A. (Cham: Springer). doi: 10.1007/10_2017_48 29470600

[B37] Cohen J. (2017). The birth of CRISPR. Science 355, 680–684. doi: 10.1126/SCIENCE.355.6326.680 28209854

[B38] Collard B. C. Y. Jahufer M. Z. Z. Brouwer J. B. Pang E. C. K. (2005). An introduction to markers, quantitative trait loci (QTL) mapping and marker-assisted selection for crop improvement: the basic concepts. Euphytica 142, 169–196. doi: 10.1007/s10681-005-1681-5

[B39] Collard B. C. Y. Mackill D. J. (2008). Marker-assisted selection: an approach for precision plant breeding in the twenty-first century. Philos. Trans. R. Soc Lond. B Biol. Sci. 363, 557–572. doi: 10.1098/rstb.2007.2170 17715053 PMC2610170

[B40] Cui L. Qiu D. Sun L. Sun Y. Ren Y. Zhang H.. (2020). Resistance to *Heterodera filipjevi* and *H. avenae* in winter wheat is conferred by different QTL. Phytopathology 110, 472–482. doi: 10.1094/PHYTO-04-19-0135-R 31433275

[B41] Curtis T. Halford N. G. (2014). Food security: the challenge of increasing wheat yield and the importance of not compromising food safety. Ann. Appl. Biol. 164, 354–372. doi: 10.1111/aab.12108 25540461 PMC4240735

[B42] Cuthbert P. A. Somers D. J. Brulé-Babel A. (2007). Mapping of Fhb2 on chromosome 6BS: a gene controlling fusarium head blight field resistance in bread wheat (*Triticum aestivum* l.). Theor. Appl. Genet. 114, 429–437. doi: 10.1007/S00122-006-0439-3 17091262

[B43] Dababat A. A. Ferney G.-B. H. Erginbas-Orakci G. Dreisigacker S. Imren M. Toktay H.. (2016). Association analysis of resistance to cereal cyst nematodes (*Heterodera avenae*) and root lesion nematodes (*Pratylenchus neglectus* and *P. thornei*) in CIMMYT advanced spring wheat lines for semi-arid conditions. Breed. Sci. 66, 15158. doi: 10.1270/jsbbs.15158 PMC5282747 28163585

[B44] Dakouri A. McCallum B. D. Walichnowski A. Z. Cloutier S. (2010). Fine mapping of the leaf rust Lr34 locus in *Triticum aestivum* (L.) and characterization of large germplasm collections support the ABC transporter as essential for gene function. Theor. Appl. Genet. 121, 373–384. doi: 10.1007/s00122-010-1316-7 20352182

[B45] Danilo B. Perrot L. Mara K. Botton E. Nogué F. Mazier M. (2019). Efficient and transgene-free gene targeting using *Agrobacterium*-mediated delivery of the CRISPR/Cas9 system in tomato. Plant Cell Rep. 38, 459–462. doi: 10.1007/S00299-019-02373-6 30649572

[B46] Delibes A. Romero D. Aguaded S. Duce A. Mena M. Lopez-Braña I.. (1993). Resistance to the cereal cyst nematode (*Heterodera avenae* woll.) transferred from the wild grassAegilops ventricosa to hexaploid wheat by a “stepping-stone” procedure. Theor. Appl. Genet. 1993 87:3 87, 402–408. doi: 10.1007/BF01184930 24190269

[B47] Dholakia B. B. Rajwade A. Hosmani P. Khan R. R. Chavan S. Reddy D. M. R.. (2013). Molecular mapping of leaf rust resistance gene Lr15 in hexaploid wheat. Mol. Breed. 31, 743–747. doi: 10.1007/s11032-012-9813-9

[B48] Dormatey R. Sun C. Ali K. Coulter J. A. Bi Z. Bai J. (2020). Gene pyramiding for sustainable crop improvement against biotic and abiotic stresses. Agronomy 10 (9), 1255. doi: 10.3390/agronomy10091255

[B49] Dumalasová V. Simmonds J. Bartoš P. Snape J. (2012). Location of genes for common bunt resistance in the European winter wheat cv. trintella. Euphytica 186, 257–264. doi: 10.1007/S10681-012-0671-7

[B50] Eastwood R. F. Lagudah E. S. Appels R. Hannah M. Kollmorgen J. F. (1991). *Triticum tauschii*: a novel source of resistance to cereal cyst nematode (*Heterodera avenae*). Aust. J. Agric. Res. 42, 69–77. doi: 10.1071/AR9910069

[B51] Eversmeyer M. G. Kramer C. L. (2000). Epidemiology of wheat leaf and stem rust in the central great plains of the USA. Annu. Rev. Phytopathol. 38, 491–513. doi: 10.1146/annurev.phyto.38.1.491 11701852

[B52] Faris J. D. Abeysekara N. S. McClean P. E. Xu S. S. Friesen T. L. (2012). Tan spot susceptibility governed by the Tsn1 locus and race-nonspecific resistance quantitative trait loci in a population derived from the wheat lines salamouni and katepwa. Mol. Breed. 30, 1669–1678. doi: 10.1007/s11032-012-9750-7

[B53] Farooq M. U. Bashir M. F. Khan M. U. S. Iqbal B. Ali Q. (2021). Role of CRISPR to improve abiotic stress tolerance in crop plants. Biol. Clin. Sci. Res. J. 2020, 69. doi: 10.54112/bcsrj.v2021i1.69

[B54] Feuillet C. Travella S. Stein N. Albar L. Nublat A. Keller B. (2003). Map-based isolation of the leaf rust disease resistance gene Lr10 from the hexaploid wheat (*Triticum aestivum* l.) genome. Proc. Natl. Acad. Sci. 100, 15253–15258. doi: 10.1073/pnas.2435133100 14645721 PMC299976

[B55] Figueroa M. Hammond-Kosack K. E. Solomon P. S. (2018). A review of wheat diseases–a field perspective. Mol. Plant Pathol. 19, 1523–1536. doi: 10.1111/mpp.12618 29045052 PMC6638159

[B56] Fischer B. M. Salakhutdinov I. Akkurt M. Eibach R. Edwards K. J. Toepfer R.. (2004). Quantitative trait locus analysis of fungal disease resistance factors on a molecular map of grapevine. Theor. Appl. Genet. 108, 501–515. doi: 10.1007/s00122-003-1445-3 14574452

[B57] Flor H. H. (1971). Current status of the gene-for-gene concept. Annu. Rev. Phytopathol. 9, 275–296. doi: 10.1146/ANNUREV.PY.09.090171.001423

[B58] Fofana B. Humphreys D. G. Cloutier S. McCartney C. A. Somers D. J. (2008). Mapping quantitative trait loci controlling common bunt resistance in a doubled haploid population derived from the spring wheat cross RL4452 x AC domain. Mol. Breed. 21, 317–325. doi: 10.1007/S11032-007-9131-9

[B59] Francia E. Tacconi G. Crosatti C. Barabaschi D. Bulgarelli D. Dall’Aglio E.. (2005). Marker assisted selection in crop plants. Plant Cell Tissue Organ Cult. 82, 317–342. doi: 10.1007/s11240-005-2387-z

[B60] Gao L. Yu H. Han W. Gao F. Liu T. Liu B.. (2014). Development of a SCAR marker for molecular detection and diagnosis of *Tilletia controversa* kühn, the causal fungus of wheat dwarf bunt. World J. Microbiol. Biotechnol. 30, 3185–3195. doi: 10.1007/s11274-014-1746-5 25269545

[B61] Ghaffary S. M. T. Faris J. D. Friesen T. L. Visser R. G. F. van der Lee T. A. J. Robert O.. (2012). New broad-spectrum resistance to *Septoria tritici* blotch derived from synthetic hexaploid wheat. Theor. Appl. Genet. 124, 125–142. doi: 10.1007/S00122-011-1692-7 21912855 PMC3249545

[B62] Ghazvini H. Hiebert C. W. Thomas J. B. Fetch T. (2013). Development of a multiple bulked segregant analysis (MBSA) method used to locate a new stem rust resistance gene (Sr54) in the winter wheat cultivar norin 40. Theor. Appl. Genet. 126, 443–449. doi: 10.1007/s00122-012-1992-6 23052026

[B63] Goates B. J. (2012). Identification of new pathogenic races of common bunt and dwarf bunt fungi, and evaluation of known races using an expanded set of differential wheat lines. Plant Dis. 96, 361–369. doi: 10.1094/PDIS-04-11-0339 30727122

[B64] Gold J. Harder D. Townley-Smith F. Aung T. Procunier J. (1999). Development of a molecular marker for rust resistance genes Sr39 and Lr35 in wheat breeding lines. Electronic J. Biotech. 2, 1–2.

[B65] Gordon T. Wang R. Hole D. Bockelman H. Michael Bonman J. Chen J. (2020). Genetic characterization and genome-wide association mapping for dwarf bunt resistance in bread wheat accessions from the USDA national small grains collection. Theor. Appl. Genet. 133, 1069–1080. doi: 10.1007/s00122-020-03532-0 31938812 PMC7021738

[B66] Goutam U. Kukreja S. Yadav R. Salaria N. Thakur K. Goyal A. K. (2015). Recent trends and perspectives of molecular markers against fungal diseases in wheat. Front. Microbiol. 6. doi: 10.3389/fmicb.2015.00861 PMC4548237 26379639

[B67] Guo J. Zhang X. Hou Y. Cai J. Shen X. Zhou T.. (2015). High-density mapping of the major FHB resistance gene Fhb7 derived from thinopyrum ponticum and its pyramiding with Fhb1 by marker-assisted selection. Theor. Appl. Genet. 128, 2301–2316. doi: 10.1007/S00122-015-2586-X 26220223

[B68] Gupta P. K. Balyan H. S. Sharma S. Kumar R. (2020). Genetics of yield, abiotic stress tolerance and biofortification in wheat (*Triticum aestivum* l.). Theor. Appl. Genet. 133, 1569–1602. doi: 10.1007/S00122-020-03583-3 32253477

[B69] Gupta S. K. Charpe A. Koul S. Haque Q. M. R. Prabhu K. V. (2006). Development and validation of SCAR markers co-segregating with an *Agropyron elongatum* derived leaf rust resistance gene Lr24 in wheat. Euphytica 150, 233–240. doi: 10.1007/s10681-006-9113-8

[B70] Gupta P. K. Langridge P. Mir R. R. (2010). Marker-assisted wheat breeding: present status and future possibilities. Mol. Breed. 26, 145–161. doi: 10.1007/S11032-009-9359-7

[B71] Gururani M. A. Venkatesh J. Upadhyaya C. P. Nookaraju A. Pandey S. K. Park S. W. (2012). Plant disease resistance genes: current status and future directions. Physiol. Mol. Plant Pathol. 78, 51–65. doi: 10.1016/J.PMPP.2012.01.002

[B72] Harshitha N. S. Sandal S. S. (2022). DNA Fingerprinting and its applications in crop improvement: a review. Pharma Innovation J. 11, 792–797.

[B73] Hasan N. Choudhary S. Naaz N. Sharma N. Laskar R. A. (2021). Recent advancements in molecular marker-assisted selection and applications in plant breeding programmes. J. Genet. Eng. Biotechnol. 19, 1–26. doi: 10.1186/s43141-021-00231-1 34448979 PMC8397809

[B74] Haugrud A. R. P. Shi G. Seneviratne S. Running K. L. Zhang Z. Singh G.. (2023). Genome-wide association mapping of resistance to the foliar diseases *septoria nodorum* blotch and tan spot in a global winter wheat collection. Theor. Appl. Genet. 135, 4169–4182. doi: 10.21203/rs.3.rs-2557769/v1 PMC10276793 37337566

[B75] Helguera M. Khan I. A. Dubcovsky J. (2000). Development of PCR markers for the wheat leaf rust resistance gene Lr47. Theor. Appl. Genet. 100, 1137–1143. doi: 10.1007/s001220051397

[B76] Helguera M. Khan I. A. Kolmer J. Lijavetzky D. Zhong-Qi L. Dubcovsky J. (2003). PCR assays for the Lr37-Yr17-Sr38 cluster of rust resistance genes and their use to develop isogenic hard red spring wheat lines. Crop Sci. 43, 1839–1847. doi: 10.2135/cropsci2003.1839

[B77] Helguera M. Vanzetti L. Soria M. Khan I. A. Kolmer J. Dubcovsky J. (2005). PCR markers for *Triticum speltoides* leaf rust resistance gene Lr51 and their use to develop isogenic hard red spring wheat lines. Crop Sci. 45, 728–734. doi: 10.2135/CROPSCI2005.0728

[B78] Herrera-Foessel S. A. Singh R. P. Huerta-Espino J. Rosewarne G. M. Periyannan S. K. Viccars L.. (2012). Lr68: a new gene conferring slow rusting resistance to leaf rust in wheat. Theor. Appl. Genet. 124, 1475–1486. doi: 10.1007/s00122-012-1802-1 22297565

[B79] Herrera-Foessel S. A. Singh R. P. Huerta-Espino J. William M. Rosewarne G. Djurle A.. (2007). Identification and mapping of Lr3 and a linked leaf rust resistance gene in durum wheat. Crop Sci. 47, 1459–1466. doi: 10.2135/CROPSCI2006.10.0663

[B80] Hickey L. T. N Hafeez A. Robinson H. Jackson S. A. Leal-Bertioli S. Tester M.. (2019). Breeding crops to feed 10 billion. Nat. Biotechnol. 37, 744–754. doi: 10.1038/s41587-019-0152-9 31209375

[B81] Hiebert C. W. Thomas J. B. McCallum B. D. Humphreys D. G. DePauw R. M. Hayden M. J.. (2010). An introgression on wheat chromosome 4DL in RL6077 (Thatcher* 6/PI 250413) confers adult plant resistance to stripe rust and leaf rust (Lr67). Theor. Appl. Genet. 121, 1083–1091. doi: 10.1007/s00122-010-1373-y 20552325

[B82] Hiebert C. W. Thomas J. B. McCallum B. D. Somers D. J. (2008). Genetic mapping of the wheat leaf rust resistance gene Lr60 (LrW2). Crop Sci. 48, 1020–1026. doi: 10.2135/cropsci2007.08.0480

[B83] Hiebert C. W. Thomas J. B. Somers D. J. McCallum B. D. Fox S. L. (2007). Erratum: microsatellite mapping of adult-plant leaf rust resistance gene Lr22a in wheat *Theor. appl. genet* . Theor. Appl. Genet. 115, 885–886. doi: 10.1007/S00122-007-0623-0 17646964

[B84] Hou W. Lu Q. Ma L. Sun X. Wang L. Nie J.. (2023). Mapping of quantitative trait loci for leaf rust resistance in the wheat population ‘Xinmai 26/Zhoumai 22’. J. Exp. Bot., erad085. doi: 10.1093/jxb/erad085 36879436

[B85] Hou S. Wu F. Wang Z. Yan N. Chen H. Li H.. (2023). Mapping stripe rust resistance QTL in ‘N2496’, a synthetic hexaploid wheat derivative. Plant Dis. 107 (2), 443–449. doi: 10.1094/PDIS-07-22-1518-RE 35802018

[B86] Huang L. Gill B. S. (2001). An RGA - like marker detects all known Lr21 leaf rust resistance gene family members in *Aegilops tauschii* and wheat. Theor. Appl. Genet. 103, 1007–1013. doi: 10.1007/S001220100701

[B87] Imbaby I. A. Mahmoud M. A. Hassan M. E. M. Abd-El-Aziz A. R. M. (2014). Identification of leaf rust resistance genes in selected egyptian wheat cultivars by molecular markers. Sci. World J. 6, 574285. doi: 10.1155/2014/574285 PMC3913395 24511291

[B88] Iqbal M. Semagn K. Randhawa H. Aboukhaddour R. Ciechanowska I. Strenzke K.. (2023). Identification and characterization of stripe rust, leaf rust, leaf spot, and common bunt resistance in spring wheat. Crop Sci. 1–19. doi: 10.1002/csc2.20953

[B89] Istifadah N. McGee P. A. (2006). Endophytic *Chaetomium globosum* reduces development of tan spot in wheat caused by *Pyrenophora tritici-repentis* . Australas. Plant Pathol. 35, 411–418. doi: 10.1071/AP06038

[B90] Jabran M. Arshad U. Aslam H. M. U. Abbas A. Haseeb A. Hussain A.. (2021). Multivariate analysis of morpho-physiological and grain yield traits in advance lines of bread wheat under different leaf rust disease regimes. Pak. J. Agric. Sci. 58, 1463–1471. doi: 10.21162/PAKJAS/21.1445

[B91] Jaganathan D. Ramasamy K. Sellamuthu G. Jayabalan S. Venkataraman G. (2018). CRISPR for crop improvement: an update review. Front. Plant Sci. 9, 985. doi: 10.3389/fpls.2018.00985 30065734 PMC6056666

[B92] Jamil S. Shahzad R. Ahmad S. Fatima R. Zahid R. Anwar M.. (2020). Role of genetics, genomics, and breeding approaches to combat stripe rust of wheat. Front. Nutr. 7, 173. doi: 10.3389/fnut.2020.580715 PMC7573350 33123549

[B93] Jevtić R. Župunski V. Lalošević M. Jocković B. Orbović B. Ilin S. (2020). Diversity in susceptibility reactions of winter wheat genotypes to obligate pathogens under fluctuating climatic conditions. Sci. Rep. 10, 19608. doi: 10.1038/s41598-020-76693-z 33184398 PMC7665191

[B94] Jiang N. Yan J. Liang Y. Shi Y. He Z. Wu Y.. (2020). Resistance genes and their interactions with bacterial blight/leaf streak pathogens (*Xanthomonas oryzae*) in rice (*Oryza sativa* l.)–an updated review. Rice 13, 3. doi: 10.1186/S12284-019-0358-Y 31915945 PMC6949332

[B95] Jing F. Jiao-Jiao X. Rin-Ming L. Yue-Qiu H. Shi-Chang X. (2013). Genetic analysis and location of gene for resistance to stripe rust in wheat international differential host strubes dickkopf. J. Genet. 92, 267–272. doi: 10.1007/s12041-013-0260-0 23970082

[B96] Karlstedt F. (2020) Identification and mapping of QTL for resistance against zymoseptoria tritici in the winter wheat accession HTRI1410 (Triticum aestivum l. subsp. spelta). dissertation. Available at: https://core.ac.uk/download/pdf/288219953.

[B97] Kassa M. T. Menzies J. G. McCartney C. A. (2014). Mapping of the loose smut resistance gene Ut6 in wheat (*Triticum aestivum* l.). Mol. Breed. 33, 569–576. doi: 10.1007/s11032-013-9973-2

[B98] Kassa M. T. You F. M. Hiebert C. W. Pozniak C. J. Fobert P. R. Sharpe A. G.. (2017). Highly predictive SNP markers for efficient selection of the wheat leaf rust resistance gene Lr16. BMC Plant Biol. 17, 45. doi: 10.1186/s12870-017-0993-7 28202046 PMC5311853

[B99] Khalil A. M. (2020). The genome editing revolution. J. Genet. Eng. Biotechnol. 18, 1–16. doi: 10.1186/s43141-020-00078-y 33123803 PMC7596157

[B100] Khan M. R. Ahamad I. Shah M. H. (2021). “Emerging important nematode problems in field crops and their management,” in Emerging trends in plant pathology. Eds. Singh K. P. Jahagirdar S. Sarma B. K. (Singapore: Springer). doi: 10.1007/978-981-15-6275-4_3

[B101] Khan M. K. Pandey A. Athar T. Choudhary S. Deval R. Gezgin S.. (2020). Fusarium head blight in wheat: contemporary status and molecular approaches. 3 Biotech. 10, 1–17. doi: 10.1007/S13205-020-2158-X/TABLES/1 32206506 PMC7080935

[B102] Kis A. Hamar É. Tholt G. Bán R. Havelda Z. (2019). Creating highly efficient resistance against wheat dwarf virus in barley by employing CRISPR/Cas9 system. Plant Biotechnol. J. 17, 1004–1006. doi: 10.1111/pbi.13077 30633425 PMC6523583

[B103] Klindworth D. L. Saini J. Long Y. Rouse M. N. Faris J. D. Jin Y.. (2017). Physical mapping of DNA markers linked to stem rust resistance gene Sr47 in durum wheat. Theor. Appl. Genet. 130, 1135–1154. doi: 10.1007/s00122-017-2875-7 28286900

[B104] Knox R. E. Campbell H. L. Clarke F. R. Menzies J. G. Popovic Z. Procunier J. D.. (2014). Quantitative trait loci for resistance in wheat (*Triticum aestivum*) to *Ustilago tritici* . Can. J. Plant Pathol. 36, 187–201. doi: 10.1080/07060661.2014.905497

[B105] Knox R. E. Campbell H. L. Depauw R. M. Gaudet D. Puchalski B. Clarke F. C. (2013). DNA Markers for resistance to common bunt in “McKenzie” wheat. Can. J. Plant Pathol. 35, 328–337. doi: 10.1080/07060661.2013.763292

[B106] Kolmer J. (2013). Leaf rust of wheat: pathogen biology, variation and host resistance. Forests 4, 70–84. doi: 10.3390/f4010070

[B107] Kolmer J. A. Anderson J. A. Flor J. M. (2010). Chromosome location, linkage with simple sequence repeat markers, and leaf rust resistance conditioned by gene Lr63 in wheat. Crop Sci. 50, 2392–2395. doi: 10.2135/cropsci2010.01.0005

[B108] Kolmer J. A. Bernardo A. Bai G. Hayden M. J. Anderson J. A. (2019). Thatcher Wheat line RL6149 carries Lr64 and a second leaf rust resistance gene on chromosome 1DS. Theor. Appl. Genet. 132, 2809–2814. doi: 10.1007/s00122-019-03389-y 31280341

[B109] Krivchenko V. I. Bakhareva Z. A. (1984). The genetic analysis of spring wheat resistance to loose smut. Genetika 20, 1337–1343.

[B110] Kumar S. Knox R. E. Singh A. K. DePauw R. M. Campbell H. L. Isidro-Sanchez J.. (2018). High-density genetic mapping of a major QTL for resistance to multiple races of loose smut in a tetraploid wheat cross. PloS One 13, e0192261. doi: 10.1371/JOURNAL.PONE.0192261 29485999 PMC5828438

[B111] Kumaran V. V. Murugasamy S. Paramasivan J. Prasad P. Kumar S. Bhardwaj S. C.. (2021). Marker assisted pyramiding of stem rust, leaf rust and powdery mildew resistance genes for durable resistance in wheat (*Triticum aestivum* l.). J. Careal Res. 13 (1), 38–48. doi: 10.25174/2582-2675/2021

[B112] Kuraparthy V. Sood S. Chhuneja P. Dhaliwal H. S. Kaur S. Bowden R. L.. (2007). A cryptic wheat–*Aegilops triuncialis* translocation with leaf rust resistance gene Lr58. Crop Sci. 47, 1995–2003. doi: 10.2135/cropsci2007.01.0038

[B113] Li Z. Lan C. He Z. Singh R. P. Rosewarne G. M. Chen X.. (2014). Overview and application of QTL for adult plant resistance to leaf rust and powdery mildew in wheat. Crop Sci. 54 (5), 1907–1925. doi: 10.2135/cropsci2014.02.0162

[B114] Li C. Wei X. Gao L. Chen W. Liu T. Liu B. (2018). ITRAQ-based proteomic analysis of wheat bunt fungi *Tilletia controversa*, *T. caries*, and *T. foetida* . Curr. Microbiol. 75, 1103–1107. doi: 10.1007/S00284-018-1490-4 29693196

[B115] Li S. Zhang C. Li J. Yan L. Wang N. Xia L. (2021). Present and future prospects for wheat improvement through genome editing and advanced technologies. Plant Commun. 2, 100211. doi: 10.1016/j.xplc.2021.100211 34327324 PMC8299080

[B116] Li J. Zhang S. Zhang R. Gao J. Qi Y. Song G.. (2021). Efficient multiplex genome editing by CRISPR/Cas9 in common wheat. Plant Biotechnol. J. 19, 427. doi: 10.1111/pbi.13508 33150679 PMC7955872

[B117] Liu J. Chang Z. Zhang X. Yang Z. Li X. Jia J.. (2013). Putative thinopyrum intermedium-derived stripe rust resistance gene Yr50 maps on wheat chromosome arm 4BL. Theor. Appl. Genet. 126, 265–274. doi: 10.1007/s00122-012-1979-3 23052018

[B118] Liu X. Xu Z. Feng B. Zhou Q. Ji G. Guo S.. (2022). Quantitative trait loci identification and breeding value estimation of grain weight-related traits based on a new wheat 50K single nucleotide polymorphism array-derived genetic map. Front. Plant Sci. 13, 967432. doi: 10.3389/fpls.2022.967432 36110352 PMC9468616

[B119] Liu S. Yu L.-X. Singh R. P. Jin Y. Sorrells M. E. Anderson J. A. (2010). Diagnostic and co-dominant PCR markers for wheat stem rust resistance genes Sr25 and Sr26. Theor. Appl. Genet. 120, 691–697. doi: 10.1007/s00122-009-1186-z 19882111

[B120] Ma J. Zhou R. Dong Y. Wang L. Wang X. Jia J. (2001). Molecular mapping and detection of the yellow rust resistance gene Yr26 in wheat transferred from *Triticum turgidum* l. using microsatellite markers. Euphytica 120, 219–226. doi: 10.1023/A:1017510331721

[B121] Ma D. Zhou X. Hou L. Bai Y. Li Q. Wang H.. (2013). Genetic analysis and molecular mapping of a stripe rust resistance gene derived from psathynrostachys huashanica keng in wheat line H9014-121-5-5-9. Mol. Breed. 32, 365–372. doi: 10.1007/s11032-013-9876-2

[B122] Mago R. Bariana H. S. Dundas I. S. Spielmeyer W. Lawrence G. J. Pryor A. J.. (2005). Development of PCR markers for the selection of wheat stem rust resistance genes Sr24 and Sr26 in diverse wheat germplasm. Theor. Appl. Genet. 111, 496–504. doi: 10.1007/s00122-005-2039-z 15918008

[B123] Mago R. Spielmeyer W. Lawrence G. Lagudah E. Ellis J. Pryor A. (2002). Identification and mapping of molecular markers linked to rust resistance genes located on chromosome 1RS of rye using wheat-rye translocation lines. Theor. Appl. Genet. 104, 1317–1324. doi: 10.1007/s00122-002-0879-3 12582587

[B124] Mago R. Tabe L. Vautrin S. Šimková H. Kubaláková M. Upadhyaya N.. (2014). Major haplotype divergence including multiple germin-like protein genes, at the wheat Sr2 adult plant stem rust resistance locus. BMC Plant Biol. 14, 379. doi: 10.1186/s12870-014-0379-z 25547135 PMC4305260

[B125] Mago R. Verlin D. Zhang P. Bansal U. Bariana H. Jin Y.. (2013). Development of wheat–*Aegilops speltoides* recombinants and simple PCR-based markers for Sr32 and a new stem rust resistance gene on the 2S# 1 chromosome. Theor. Appl. Genet. 126, 2943–2955. doi: 10.1007/s00122-013-2184-8 23989672

[B126] Majumdar R. Rajasekaran K. Cary J. W. (2017). RNA Interference (RNAi) as a potential tool for control of mycotoxin contamination in crop plants: concepts and considerations. Front. Plant Sci. 8. doi: 10.3389/FPLS.2017.00200/FULL PMC5306134 28261252

[B127] Mateos-Hernandez M. Singh R. P. Hulbert S. H. Bowden R. L. Huerta-Espino J. Gill B. S.. (2006). Targeted mapping of ESTs linked to the adult plant resistance gene Lr46 in wheat using synteny with rice. Funct. Integr. Genom. 6, 122–131. doi: 10.1007/s10142-005-0017-9 16374594

[B128] McCartney C. A. Brûlé-Babel A. L. Lamari L. Somers D. J. (2003). Chromosomal location of a race-specific resistance gene to *Mycosphaerella graminicola* in the spring wheat ST6. Theor. Appl. Genet. 107, 1181–1186. doi: 10.1007/S00122-003-1359-0 12898022

[B129] McLaughlin J. E. Darwish N. I. Garcia-Sanchez J. Tyagi N. Trick H. N. McCormick S.. (2021). A lipid transfer protein has antifungal and antioxidant activity and suppresses fusarium head blight disease and DON accumulation in transgenic wheat. Phytopathology 111, 671–683. doi: 10.1094/PHYTO-04-20-0153-R 32896217

[B130] Mebrate S. A. Dehne H. Pillen K. Oerke E. (2008). Postulation of seedling leaf rust resistance genes in selected Ethiopian and German bread wheat cultivars. Crop Sci. 48, 507–516. doi: 10.2135/cropsci2007.03.0173

[B131] Menzies J. G. Knox R. E. Popovic Z. Procunier J. D. (2006). Common bunt resistance gene Bt10 located on wheat chromosome 6D. Can. J. Plant Sci. 86, 1409–1412. doi: 10.4141/P06-106

[B132] Metakovsky E. Pascual L. Vaccino P. Melnik V. Rodriguez-Quijano M. Popovych Y.. (2021). Heteroalleles in common wheat: multiple differences between allelic variants of the gli-B1 locus. Int. J. Mol. Sci. 22, 1832. doi: 10.3390/ijms22041832 33673225 PMC7917834

[B133] Miedaner T. Korzun V. (2012). Marker-assisted selection for disease resistance in wheat and barley breeding. Phytopathology 102, 560–566. doi: 10.1094/PHYTO-05-11-0157 22568813

[B134] Monteiro F. Nishimura M. T. (2018). Structural, functional, and genomic diversity of plant NLR proteins: an evolved resource for rational engineering of plant immunity. Annu. Rev. Phytopathol. 56, 243–267. doi: 10.1146/ANNUREV-PHYTO-080417-045817 29949721

[B135] Montes M. J. Andrés M. F. Sin E. López-Braña I. Martín-Sánchez J. A. Romero M. D.. (2008). Cereal cyst nematode resistance conferred by the Cre7 gene from *Aegilops triuncialis* and its relationship with cre genes from Australian wheat cultivars. Genome 51, 315–319. doi: 10.1139/G08-015 18438434

[B136] Montesclaros J. M. L. Teng P. S. (2021). Agriculture and food security in Asia, In: Pulhin J. M. Inoue M. Shaw R. (eds) climate change, disaster risks, and human security. Disaster Risk Reduction (Singapore: Springer). doi: 10.1007/978-981-15-8852-5_7

[B137] Mores A. Borrelli G. M. Laidò G. Petruzzino G. Pecchioni N. Amoroso L. G. M.. (2021). Genomic approaches to identify molecular bases of crop resistance to diseases and to develop future breeding strategies. Int. J. Mol. Sci. 22, 5423. doi: 10.3390/ijms22115423 34063853 PMC8196592

[B138] Mourad A. M. I. Sallam A. Belamkar V. Mahdy E. Bakheit B. Abo El-Wafaa A.. (2018). Genetic architecture of common bunt resistance in winter wheat using genome-wide association study. BMC Plant Biol. 18, 280. doi: 10.1186/s12870-018-1435-x 30424724 PMC6234641

[B139] Muellner A. E. Buerstmayr M. Eshonkulov B. Hole D. Michel S. Hagenguth J. F.. (2021). Comparative mapping and validation of multiple disease resistance QTL for simultaneously controlling common and dwarf bunt in bread wheat. Theor. Appl. Genet. 134, 489–503. doi: 10.1007/s00122-020-03708-8 33120433 PMC7843488

[B140] Muellner A. E. Eshonkulov B. Hagenguth J. Pachler B. Michel S. Buerstmayr M.. (2020). Genetic mapping of the common and dwarf bunt resistance gene Bt12 descending from the wheat landrace PI119333. Euphytica 216, 1–15. doi: 10.1007/s10681-020-02614-w

[B141] Muhammad A. Hu W. Li Z. Li J. Xie G. Wang J.. (2020). Appraising the genetic architecture of kernel traits in hexaploid wheat using GWAS. Int. J. Mol. Sci. 21, 5649. doi: 10.3390/ijms21165649 32781752 PMC7460857

[B142] Muhu-Din Ahmed H. G. Naeem M. Zeng Y. Rashid M. A. R. Ullah A. Saeed A.. (2022). Genome-wide association mapping for high temperature tolerance in wheat through 90k SNP array using physiological and yield traits. PloS One 17, e0262569. doi: 10.1371/JOURNAL.PONE.0262569 35030233 PMC8759701

[B143] Mulki M. A. Jighly A. Ye G. Emebiri L. C. Moody D. Ansari O.. (2013). Association mapping for soilborne pathogen resistance in synthetic hexaploid wheat. Mol. Breed. 31, 299–311. doi: 10.1007/S11032-012-9790-Z

[B144] Muqaddasi Q. H. Kamal R. Mirdita V. Rodemann B. Ganal M. W. Reif J. C.. (2021). Genome-wide association studies and prediction of tan spot (*Pyrenophora tritici*-repentis) infection in european winter wheat *via* different marker platforms. Genes 12 (4), 490. doi: 10.3390/genes12040490 33801723 PMC8103242

[B145] Murray G. Brennan J. P. (2009). Estimating disease losses to the Australian wheat industry. Australas. Plant Pathol. 38, 558–570. doi: 10.1071/AP09053

[B146] Naik B. K. Sharma J. B. Sivasamy M. Prabhu K. Tomar R. S. Tomar S. M. S. (2015). Molecular mapping and validation of the microsatellite markers linked to the secale cereale-derived leaf rust resistance gene Lr45 in wheat. Mol. Breed. 35, 1–10. doi: 10.1007/s11032-015-0234-4

[B147] Nelson J. C. Singh R. P. Autrique J. E. Sorrells M. E. (1997). Mapping genes conferring and suppressing leaf rust resistance in wheat. Crop Sci. 37, 1928–1935. doi: 10.2135/cropsci1997.0011183X003700060043x

[B148] Neu C. Stein N. Keller B. (2002). Genetic mapping of the Lr20 Pm1 resistance locus reveals suppressed recombination on chromosome arm 7AL in hexaploid wheat. Genome 45, 737–744. doi: 10.1139/g02-040 12175077

[B149] Nigus M. Shimelis H. Mathew I. Abady S. (2022). Wheat production in the highlands of Eastern Ethiopia: opportunities, challenges and coping strategies of rust diseases. Acta Agric. Scand. B Soil Plant Sci. 72 (1), 563–575. doi: 10.1080/09064710.2021.2022186

[B150] Niu Z. Klindworth D. L. Yu G. L Friesen T. Chao S. Jin Y.. (2014). Development and characterization of wheat lines carrying stem rust resistance gene Sr43 derived from *Thinopyrum ponticum* . Theor. Appl. Genet. 127, 969–980. doi: 10.1007/s00122-014-2272-4 24504553

[B151] Ogbonnaya F. C. Eastwood R. F. Lagudah E. (2009). “Identification and utilisation of genes for cereal cyst nematode (Heterodera avenae) resistance in wheat: the Australian experience,” in Cereal cyst nematodes: status, research and outlook. Eds. Riley I. T. Nicol M. J. Dababat A. A. (Ankara, Turkey: CIMMYT), 166–171.

[B152] Ogbonnaya F. C. Seah S. Delibes A. Jahier J. López-Braña I. Eastwood R. F.. (2001). Molecular-genetic characterisation of a new nematode resistance gene in wheat. Theor. Appl. Genet. 102, 623–629. doi: 10.1007/S001220051689

[B153] Paix A. Folkmann A. Seydoux G. (2017). Precision genome editing using CRISPR-Cas9 and linear repair templates in *C. elegans* . Methods 121, 86–93. doi: 10.1016/j.ymeth.2017.03.023 28392263 PMC6788293

[B154] Park R. Fetch T. Hodson D. Jin Y. Nazari K. Prashar M.. (2011). International surveillance of wheat rust pathogens: progress and challenges. Euphytica 179, 109–117. doi: 10.1007/S10681-011-0375-4

[B155] Periyannan S. Bansal U. Bariana H. Deal K. Luo M.-C. Dvorak J.. (2014). Identification of a robust molecular marker for the detection of the stem rust resistance gene Sr45 in common wheat. Theor. Appl. Genet. 127, 947–955. doi: 10.1007/s00122-014-2270-6 24469473

[B156] Periyannan S. K. Bansal U. K. Bariana H. S. Pumphrey M. Lagudah E. S. (2011). A robust molecular marker for the detection of shortened introgressed segment carrying the stem rust resistance gene Sr22 in common wheat. Theor. Appl. Genet. 122, 1–7. doi: 10.1007/s00122-010-1417-3 20680609

[B157] Phipps S. N. Burke A. B. Balow K. Smith J. Murray T. Carter A. H. (2022). Identification of snow mold tolerance QTL in a landrace winter wheat using linkage mapping. Crop Sci. 62, 1415–1429. doi: 10.1002/csc2.20745

[B158] Phuke R. M. He X. Juliana P. Bishnoi S. K. Singh G. P. Kabir M. R.. (2020). Association mapping of seedling resistance to tan spot (*Pyrenophora tritici-repentis* race 1) in CIMMYT and south Asian wheat germplasm. Front. Plant Sci. 11, 1309. doi: 10.3389/fpls.2020.01309 32983199 PMC7483578

[B159] Qi L. Cao M. Chen P. Li W. Liu D. (1996). Identification, mapping, and application of polymorphic DNA associated with resistance gene Pm21 of wheat. Genome 39, 191–197. doi: 10.1139/G96-025 18469886

[B160] Rahman M. S. Linsell K. J. Taylor J. D. Hayden M. J. Collins N. C. Oldach K. H. (2020). Fine mapping of root lesion nematode (*Pratylenchus thornei*) resistance loci on chromosomes 6D and 2B of wheat. Theor. Appl. Genet. 133, 635–652. doi: 10.1007/s00122-019-03495-x 31813000

[B161] Rajesh M. K. Ramesh S. V. Perera L. Manickavelu A. (2021). Quantitative Trait Loci (QTL) and association mapping for major agronomic traits. In: Rajesh M. K. Ramesh S. V. Perera L. Kole C.. (eds) The coconut genome. Compendium of Plant Genomes (Cham: Springer), doi: 10.1007/978-3-030-76649-8_6

[B162] Ram B. Singh K. P. (2004). Smuts of wheat: a review. Indian Phytopath 57, 125–134.

[B163] Raman R. Milgate A. W. Imtiaz M. Tan M. K. Raman H. Lisle C.. (2009). Molecular mapping and physical location of major gene conferring seedling resistance to *Septoria tritici* blotch in wheat. Mol. Breed. 24, 153–164. doi: 10.1007/S11032-009-9280-0

[B164] Ramirez-Gonzalez R. H. Segovia V. Bird N. Fenwick P. Holdgate S. Berry S.. (2015). RNA-Seq bulked segregant analysis enables the identification of high-resolution genetic markers for breeding in hexaploid wheat. Plant Biotechnol. J. 13, 613–624. doi: 10.1111/PBI.12281 25382230

[B165] Randhawa H. S. Asif M. Pozniak C. Clarke J. M. Graf R. J. Fox S. L.. (2013). Application of molecular markers to wheat breeding in c anada. Plant Breed. 132 (5), 458–471. doi: 10.1111/pbr.12057

[B166] Randhawa M. Bansal U. Valárik M. Klocová B. Doležel J. Bariana H. (2014). Molecular mapping of stripe rust resistance gene Yr51 in chromosome 4AL of wheat. Theor. Appl. Genet. 127, 317–324. doi: 10.1007/s00122-013-2220-8 24185819

[B167] Rani K. Raghu B. R. Jha S. K. Agarwal P. Mallick N. Niranjana M.. (2020). A novel leaf rust resistance gene introgressed from aegilops markgrafii maps on chromosome arm 2AS of wheat. Theor. Appl. Genet. 133, 2685–2694. doi: 10.1007/s00122-020-03625-w 32507913

[B168] Rao M. J. Wang L. (2021). CRISPR/Cas9 technology for improving agronomic traits and future prospective in agriculture. Planta 254, 1–16. doi: 10.1007/S00425-021-03716-Y 34498163

[B169] Raupp W. J. Brown-Guedira G. L. Gill B. S. (2001). Cytogenetic and molecular mapping of the leaf rust resistance gene Lr39 in wheat. Theor. Appl. Genet. 102, 347–352. doi: 10.1007/s001220051652

[B170] Reynolds M. P. Borlaug N. E. (2006). Impacts of breeding on international collaborative wheat improvement. J. Agric. Sci. 144 (1), 3–17. doi: 10.1017/S0021859606005867

[B171] Robert O. Abelard C. Dedryver F. (1999). Identification of molecular markers for the detection of the yellow rust resistance gene Yr17 in wheat. Mol. Breed. 5, 167–175. doi: 10.1023/A:1009672021411

[B172] Robinson N. A. Sheedy J. G. Macdonald B. J. Kirsty, Owen J. Thompson J. P. (2019). Tolerance of wheat cultivars to root-lesion nematode (*Pratylenchus thornei*) assessed by normalised difference vegetation index is predictive of grain yield. Ann. Appl. Biol. 174, 388–401. doi: 10.1111/aab.12504

[B173] Romero F. M. Gatica-Arias A. (2019). CRISPR/Cas9: development and application in rice breeding. Rice Sci. 26, 265–281. doi: 10.1016/j.rsci.2019.08.001

[B174] Rouse M. N. Nava I. C. Chao S. Anderson J. A. Jin Y. (2012). Identification of markers linked to the race Ug99 effective stem rust resistance gene Sr28 in wheat (*Triticum aestivum* l.). Theor. Appl. Genet. 125, 877–885. doi: 10.1007/s00122-012-1879-6 22584633

[B175] Rush C. M. Stein J. M. Bowden R. L. Riemenschneider R. Boratynski T. Royer M. H. (2005). Status of karnal bunt of wheat in the united states 1996 to 2004. Plant Dis. 89, 212–223. doi: 10.1094/PD-89-0212 30795341

[B176] Savadi S. Prasad P. Kashyap P. L. Bhardwaj S. C. (2018). Molecular breeding technologies and strategies for rust resistance in wheat (*Triticum aestivum*) for sustained food security. Plant Pathol. 67, 771–791. doi: 10.1111/ppa.12802

[B177] Sawyer C. R. Labbé J. L. (2021). Small RNAs at the interface of the plant-fungal interactions. Fungal Genom. Biol. 11, 1–4.

[B178] Schaefer L. K. Parlange F. Buchmann G. Jung E. Wehrli A. Herren G.. (2020). Cross-kingdom RNAi of pathogen effectors leads to quantitative adult plant resistance in wheat. Front. Plant Sci. 11. doi: 10.3389/FPLS.2020.00253/FULL PMC7076181 32211008

[B179] Schnurbusch T. Paillard S. Fossati D. Messmer M. Schachermayr G. Winzeler M.. (2003). Detection of QTLs for stagonospora glume blotch resistance in Swiss winter wheat. Theor. Appl. Genet. 107, 1226–1234. doi: 10.1007/S00122-003-1372-3 12928778

[B180] Seid A. İmren M. Ali M. A. Toumi F. Paulitz T. Dababat A. A. (2021). Genetic resistance of wheat towards plant-parasitic nematodes: current status and future prospects. Biotech. Stud. 30, 43–62. doi: 10.38042/biotechstudies.944678

[B181] Shan Q. Wang Y. Li J. Gao C. (2014). Genome editing in rice and wheat using the CRISPR/Cas system. Nat. Protoc. 9, 2395–2410. doi: 10.1038/nprot.2014.157 25232936

[B182] Singh S. Bowden R. L. (2011). Molecular mapping of adult-plant race-specific leaf rust resistance gene Lr12 in bread wheat. Mol. Breed. 28, 137–142. doi: 10.1007/s11032-010-9467-4

[B183] Singh R. P. Hodson D. P. Huerta-Espino J. Jin Y. Bhavani S. Njau P.. (2011). The emergence of Ug99 races of the stem rust fungus is a threat to world wheat production. Annu. Rev. Phytopathol. 49, 465–481. doi: 10.1146/annurev-phyto-072910-095423 21568701

[B184] Singh A. Knox R. E. DePauw R. M. Singh A. K. Cuthbert R. D. Kumar S.. (2016). Genetic mapping of common bunt resistance and plant height QTL in wheat. Theor. Appl. Genet. 129, 243–256. doi: 10.1007/S00122-015-2624-8 26520114

[B185] Singh A. Pallavi J. K. Gupta P. Prabhu K. V. (2012). Identification of microsatellite markers linked to leaf rust resistance gene Lr25 in wheat. J. Appl. Genet. 53, 19–25. doi: 10.1007/s13353-011-0070-0 22033869

[B186] Sinha P. Chen X. (2021). Potential infection risks of the wheat stripe rust and stem rust pathogens on barberry in Asia and southeastern Europe. Plants 10, 957. doi: 10.3390/plants10050957 34064962 PMC8151100

[B187] Smiley R. W. Dababat A. A. Iqbal S. Jones M. G. Maafi Z. T. Peng D.. (2017). Cereal cyst nematodes: a complex and destructive group of *Heterodera* species. Plant Dis. 101 (10), 1692–1720. doi: 10.1094/PDIS-03-17-0355-FE 30676930

[B188] Sohail Y. Bansal U. Bariana H. Chhuneja P. Mumtaz A. Rattu A.. (2014). Identification of a co-dominant eSTS marker linked with leaf rust resistance gene’Lr28’in wheat (‘*Triticum aestivum*’L.). Aust. J. Crop Sci. 8, 1210–1215.

[B189] Steffan P. M. Torp A. M. Borgen A. Backes G. Rasmussen S. K. (2017). Mapping of common bunt resistance gene Bt9 in wheat. Theor. Appl. Genet. 130, 1031–1040. doi: 10.1007/S00122-017-2868-6 28238022 PMC5395592

[B190] Sthapit J. Newcomb M. Bonman J. M. Chen X. See D. R. (2014). Genetic diversity for stripe rust resistance in wheat landraces and identification of accessions with resistance to stem rust and stripe rust. Crop Sci. 54, 2131–2139. doi: 10.2135/CROPSCI2013.07.0438

[B191] Sthapit Kandel J. Krishnan V. Jiwan D. Chen X. Skinner D. Z. See D. R. (2017). Mapping genes for resistance to stripe rust in spring wheat landrace PI 480035. PloS One 12, e0177898. doi: 10.1371/journal.pone.0177898 28542451 PMC5438115

[B192] Sun X. Bai G. Carver B. F. (2009). Molecular markers for wheat leaf rust resistance gene Lr41. Mol. Breed. 23, 311–321. doi: 10.1007/s11032-008-9237-8

[B193] Sun G. L. Fahima T. Korol A. B. Turpeinen T. Grama A. Ronin Y. I.. (1997). Identification of molecular markers linked to the Yr15 stripe rust resistance gene of wheat originated in wild emmer wheat, *Triticum dicoccoides* . Theor. Appl. Genet. 95, 622–628. doi: 10.1007/s001220050604

[B194] Sun N. Zhao H. (2013). Transcription activator-like effector nucleases (TALENs): a highly efficient and versatile tool for genome editing. Biotechnol. Bioeng. 110, 1811–1821. doi: 10.1002/BIT.24890 23508559

[B195] Swamy B. P. M. Sarla N. (2008). Yield-enhancing quantitative trait loci (QTLs) from wild species. Biotechnol. Adv. 26, 106–120. doi: 10.1016/j.biotechadv.2007.09.005 17949936

[B196] Tabib Ghaffary S. M. Robert O. Laurent V. Lonnet P. Margalé E. van der Lee T. A. J.. (2011). Genetic analysis of resistance to *Septoria tritici* blotch in the French winter wheat cultivars balance and Apache. Theor. Appl. Genet. 123, 741–754. doi: 10.1007/S00122-011-1623-7 21655994 PMC3155673

[B197] Talebi R. Mahboubi M. Naji A. M. Mehrabi R. (2023). Physiological specialization of *Puccinia triticina* and genome-wide association mapping provide insights into the genetics of wheat leaf rust resistance in Iran. Sci. Rep. 13 (1), 4398. doi: 10.1038/s41598-023-31559-y 36927878 PMC10020449

[B198] Taning C. N. T. Arpaia S. Christiaens O. Dietz-Pfeilstetter A. Jones H. Mezzetti B.. (2020). RNA-Based biocontrol compounds: current status and perspectives to reach the market. Pest Manage. Sci. 76, 841–845. doi: 10.1002/ps.5686 31743573

[B199] Tehseen M. M. Tonk F. A. Tosun M. Randhawa H. S. Kurtulus E. Ozseven I.. (2022). QTL mapping of adult plant resistance to stripe rust in a doubled haploid wheat population. Front. Genet. 13, 900558. doi: 10.3389/fgene.2022.900558 35646084 PMC9131033

[B200] Terracciano I. Maccaferri M. Bassi F. Mantovani P. Sanguineti M. C. Salvi S.. (2013). Development of COS-SNP and HRM markers for high-throughput and reliable haplotype-based detection of Lr14a in durum wheat (*Triticum durum* desf.). Theor. Appl. Genet. 126, 1077–1101. doi: 10.1007/s00122-012-2038-9 23292293

[B201] Thambugala D. Menzies J. G. Knox R. E. Campbell H. L. McCartney C. A. (2020). Genetic analysis of loose smut (*Ustilago tritici*) resistance in sonop spring wheat. BMC Plant Biol. 20, 314. doi: 10.1186/s12870-020-02525-x 32620083 PMC7333308

[B202] Thompson J. P. Brennan P. S. Clewett T. G. Sheedy J. G. Seymour N. P. (1999). Progress in breeding wheat for tolerance and resistance to root-lesion nematode (*Pratylenchus thornei*). Australas. Plant Pathol. 28, 45–52. doi: 10.1071/AP99006

[B203] Thomson M. J. (2014). High-throughput SNP genotyping to accelerate crop improvement. Plant Breed. Biotechnol. 2, 195–212. doi: 10.9787/PBB.2014.2.3.195

[B204] Todorovska E. Christov N. Slavov S. Christova P. Vassilev D. (2009). Biotic stress resistance in wheat–breeding and genomic selection implications. Biotechnol. Biotechnol. Equip 23, 1417–1426. doi: 10.2478/V10133-009-0006-6

[B205] Toktay H. McIntyre C. L. Nicol J. M. Ozkan H. Elekcioglu H. I. (2006). Identification of common root-lesion nematode (*Pratylenchus thornei* sher et Allen) loci in bread wheat. Genome 49 (10), 1319–1323. doi: 10.1139/g06-090 17213914

[B206] Toor A. Bansal U. Bariana H. (2013). Mapping of flag smut resistance in common wheat. Mol. Breed. 32, 699–707. doi: 10.1007/s11032-013-9903-3

[B207] Toor A. K. Bariana H. S. (2012). “Flag smut of wheat-pathogen biology and host resistance,” in Disease resistance in wheat, CABI Plant Protection Series. CABI International; 295–303. doi: 10.1079/9781845938185.0295

[B208] Townsend J. A. Wright D. A. Winfrey R. J. Fu F. Maeder M. L. Joung J. K.. (2009). High-frequency modification of plant genes using engineered zinc-finger nucleases. Nature 459, 442–445. doi: 10.1038/NATURE07845 19404258 PMC2743854

[B209] Tsilo T. J. Jin Y. Anderson J. A. (2007). Microsatellite markers linked to stem rust resistance allele Sr9a in wheat. Crop Sci. 47 (5), 2013–2020. doi: 10.2135/cropsci2007.02.0087

[B210] Tyagi S. Kumar R. Kumar V. Won S. Y. Shukla P. (2021). Engineering disease resistant plants through CRISPR-Cas9 technology. GM Crops Food 12, 125–144. doi: 10.1080/21645698.2020.1831729 33079628 PMC7583490

[B211] Ullah M. Xia L. Xie S. Applied S. S.-B. (2020). CRISPR/Cas9-based genome engineering: a new breakthrough in the genetic manipulation of filamentous fungi. Biotechnol. Appl. Biochem. 67, 835–851. doi: 10.1002/bab.2077 33179815

[B212] Vanzetti L. S. Campos P. Demichelis M. Lombardo L. A. Aurelia P. R. Vaschetto L. M.. (2011). Identification of leaf rust resistance genes in selected argentinean bread wheat cultivars by gene postulation and molecular markers. Electron. J. Biotechnol. 14 (3), 9–9. doi: 10.2225/vol14-issue3-fulltext-14

[B213] Vega-Arreguín J. C. Shimada-Beltrán H. Sevillano-Serrano J. Moffett P. (2017). Non-host plant resistance against phytophthora capsici is mediated in part by members of the I2 r gene family in nicotiana spp. Front. Plant Sci. 8, 205. doi: 10.3389/fpls.2017.00205 28261255 PMC5309224

[B214] Verges V. L. Brown-Guedira G. L. van Sanford D. A. (2021). Genome-wide association studies combined with genomic selection as a tool to increase fusarium head blight resistance in wheat. Crop Breed. Genet. Genom. 3 (4), e210007. doi: 10.20900/cbgg20210007

[B215] Vinod K. K. (2009). “Genetic mapping of quantitative trait loci and marker assisted selection in plantation crops,” In: Proceedings of the training programme on “In vitro Techniques in Plantation Crops”. Central Plantation Crops Research Institute, Kasaragod, India. pp. 111–132. Available at: http://kkvinod.webs.com.

[B216] Wan A. Muleta K. T. Zegeye H. Hundie B. Pumphrey M. O. Chen X. (2017). Virulence characterization of wheat stripe rust fungus *Puccinia striiformis* f. sp. *tritici* in Ethiopia and evaluation of Ethiopian wheat germplasm for resistance to races of the pathogen from Ethiopia and the united states. Plant Dis. 101, 73–80. doi: 10.1094/PDIS-03-16-0371-RE 30682307

[B217] Wang Y. Cheng X. Shan Q. Zhang Y. Liu J. Gao C.. (2014). Simultaneous editing of three homoeoalleles in hexaploid bread wheat confers heritable resistance to powdery mildew. Nat. Biotech. 32:9 32, 947–951. doi: 10.1038/nbt.2969 25038773

[B218] Wang R. Gordon T. Hole D. Zhao W. Isham K. Bonman J. M.. (2019). Identification and assessment of two major QTLs for dwarf bunt resistance in winter wheat line ‘IDO835.’. Theor. Appl. Genet. 132, 2755–2766. doi: 10.1007/s00122-019-03385-2 31240345

[B219] Wang L. Ma J. Zhou R. Wang X. Jia J. (2002). Molecular tagging of the yellow rust resistance gene Yr10 in common wheat, P.I.178383 (*Triticum aestivum* l.). Euphytica 124, 71–73. doi: 10.1023/A:1015689817857

[B220] Wang W. Pan Q. He F. Akhunova A. Chao S. Trick H.. (2018). Transgenerational CRISPR-Cas9 activity facilitates multiplex gene editing in allopolyploid wheat. CRISPR J. 1, 65–74. doi: 10.1089/CRISPR.2017.0010 30627700 PMC6319321

[B221] Wang C. Zhang Y. Han D. Kang Z. Li G. Cao A.. (2008). SSR and STS markers for wheat stripe rust resistance gene Yr26. Euphytica 159, 359–366. doi: 10.1007/s10681-007-9524-1

[B222] Wellings C. R. (2011). Global status of stripe rust: a review of historical and current threats. Euphytica 179, 129–141. doi: 10.1007/S10681-011-0360-Y

[B223] Wilcoxson R. D. (1996). Bunt and smut diseases of wheat: concepts and methods of disease management. Vol. 4 (Idaho, Mexico, USA: CIMMYT).

[B224] Williams K. J. Lewis J. G. Bogacki P. Pallotta M. A. Willsmore K. L. Kuchel H.. (2003). Mapping of a QTL contributing to cereal cyst nematode tolerance and resistance in wheat. Aust. J. Agric. Res. 54, 731–737. doi: 10.1071/AR02225

[B225] Williams K. J. Taylor S. P. Bogacki P. Pallotta M. Bariana H. S. Wallwork H. (2002). Mapping of the root lesion nematode (*Pratylenchus neglectus*) resistance gene Rlnn1 in wheat. Theor. Appl. Genet. 104, 874–879. doi: 10.1007/s00122-001-0839-3 12582649

[B226] Williams K. J. Willsmore K. L. Olson S. Matic M. Kuchel H. (2006). Mapping of a novel QTL for resistance to cereal cyst nematode in wheat. Theor. Appl. Genet. 112, 1480–1486. doi: 10.1007/s00122-006-0251-0 16538511

[B227] Wu P. Xie J. Hu J. Qiu D. Liu Z. Li J.. (2018). Development of molecular markers linked to powdery mildew resistance gene Pm4b by combining SNP discovery from transcriptome sequencing data with bulked segregant analysis (BSR-seq) in wheat. Front. Plant Sci. 9. doi: 10.3389/FPLS.2018.00095/FULL PMC5817070 29491869

[B228] Würschum T. Liu W. Gowda M. Maurer H. P. Fischer S. Schechert A.. (2012). Comparison of biometrical models for joint linkage association mapping. Heredity 108 (3), 332–340. doi: 10.1038/hdy.2011.78 21878984 PMC3282402

[B229] Xu L. S. Wang M. N. Cheng P. Kang Z. S. Hulbert S. H. Chen X. M. (2013). Molecular mapping of Yr53, a new gene for stripe rust resistance in durum wheat accession PI 480148 and its transfer to common wheat. Theor. Appl. Genet. 126, 523–533. doi: 10.1007/s00122-012-1998-0 23090143

[B230] Yadaw R. B. Dixit S. Raman A. Mishra K. K. Vikram P. Swamy B. P. M.. (2013). A QTL for high grain yield under lowland drought in the background of popular rice variety sabitri from Nepal. Field Crops Res. 144, 281–287. doi: 10.1016/j.fcr.2013.01.019

[B231] Yan G. Chen X. Line R. Wellings C. (2003). Resistance gene-analog polymorphism markers co-segregating with the Yr5 gene for resistance to wheat stripe rust. Theor. Appl. Genet. 106, 636–643. doi: 10.1007/s00122-002-1109-8 12595992

[B232] Yan J. Yang Z. Cheng J. (2011). Resistance gene analogs markers linked to the stripe rust resistance gene YrH52 derived from wild emmer wheat (*Triticum dicoccoides*). J. Triticeae Crops 31, 590–597.

[B233] Yang N. Yan L. Zheng Z. Zhang Y. Zhan H. Tian Y.. (2022). Editing gene families by CRISPR/Cas9: accelerating the isolation of multiple transgene-free null mutant combinations with much reduced labor-intensive analysis. Plant Biotech. J. 20, 241–243. doi: 10.1111/pbi.13744 PMC8753350 34726841

[B234] Yao Z. Qin D. Chen D. Liu C. Chen W. Liu T.. (2019). Development of ISSR-derived SCAR marker and SYBR green I real-time PCR method for detection of teliospores of *Tilletia laevis* kühn. Sci. Rep. 9, 17651. doi: 10.1038/s41598-019-54163-5 31776416 PMC6881473

[B235] Ye G. Smith K. F. (2008). Marker-assisted gene yramiding for inbred line development: basic principles and practical guidelines. Int. J. Plant Breed. 2 (1), 1–10.

[B236] Young N. D. (1996). QTL mapping and quantitative disease resistance in plants. Ann. Rev. Phytopathol. 34 (1), 479–501. doi: 10.1146/annurev.phyto.34.1.479 15012553

[B237] Yu L.-X. Barbier H. Rouse M. N. Singh S. Singh R. P. Bhavani S.. (2014). A consensus map for Ug99 stem rust resistance loci in wheat. Theor. Appl. Genet. 127, 1561–1581. doi: 10.1007/s00122-014-2326-7 24903979 PMC4072096

[B238] Yuan C. Wu J. Yan B. Hao Q. Zhang C. Lyu B.. (2018). Remapping of the stripe rust resistance gene Yr10 in common wheat. Theor. Appl. Genet. 131, 1253–1262. doi: 10.1007/s00122-018-3075-9 29476226

[B239] Zaidi S. S. E. A. Tashkandi M. Mansoor S. Mahfouz M. M. (2016). Engineering plant immunity: using CRISPR/Cas9 to generate virus resistance. Front. Plant Sci. 7. doi: 10.3389/FPLS.2016.01673/FULL PMC5099147 27877187

[B240] Zhang Y. Bai Y. Wu G. Zou S. Chen Y. Gao C.. (2017). Simultaneous modification of three homoeologs of TaEDR1 by genome editing enhances powdery mildew resistance in wheat. Plant J. 91, 714–724. doi: 10.1111/tpj.13599 28502081

[B241] Zhang M. Chen W. Q. Liu D. Liu T. G. Gao L. Shu K. (2012). Identification of a specific SCAR marker for detection of *Tilletia foetida* (Wall) liro pathogen of wheat. Russ. J. Genet. 48, 663–666. doi: 10.1134/S1022795412050237 22946337

[B242] Zhang R. Feng Y. Li H. Yuan H. Dai J. Cao A.. (2016). Cereal cyst nematode resistance gene CreV effective against *Heterodera filipjevi* transferred from chromosome 6VL of *Dasypyrum villosum* to bread wheat. Mol. Breed. 36, 1–11. doi: 10.1007/S11032-016-0549-9

[B243] Zhang Y. Li D. Zhang D. Zhao X. Cao X. Dong L.. (2018). Analysis of the functions of TaGW2 homoeologs in wheat grain weight and protein content traits. Plant J. 94, 857–866. doi: 10.1111/TPJ.13903 29570880

[B244] Zhang W. Olson E. Saintenac C. Rouse M. Abate Z. Jin Y.. (2010). Genetic maps of stem rust resistance gene Sr35 in diploid and hexaploid wheat. Crop Sci. 50, 2464–2474. doi: 10.2135/cropsci2010.04.0202

[B245] Zhang D. Zhang Z. Unver T. Zhang B. (2021). CRISPR/Cas: a powerful tool for gene function study and crop improvement. J. Adv. Res. 29, 207–221. doi: 10.1016/j.jare.2020.10.003 33842017 PMC8020163

[B246] Zhang L. Zhou H. Wei F. Cheng Z. Yan A. Wang D. (2011). Construction of yeast two-hybrid cDNA libraries for wheat near-isogenic line TcLr19 under the stress of *Puccinia recondita* and its preliminary appreciation. Front. Agric. China 5, 450–455. doi: 10.1007/s11703-011-1123-1

[B247] Zheng S. Wu Y. Zhou M. Zeng L. Liu R. Li Y.. (2020). Characterization and diagnostic marker development for Yr28-rga1 conferring stripe rust resistance in wheat. Eur. J. Plant Pathol. 156, 623–634. doi: 10.1007/s10658-019-01912-x

[B248] Zhou X. Li X. Han D. Yang S. Kang Z. Ren R. (2022). Genome-wide QTL mapping for stripe rust resistance in winter wheat pindong 34 using a 90K SNP array. Front. Plant Sci. 13, 4. doi: 10.3389/fpls.2022.932762 PMC9296828 35873978

[B249] Zhou Y. Ren Y. Lillemo M. Yao Z. Zhang P. Xia X.. (2014). QTL mapping of adult-plant resistance to leaf rust in a RIL population derived from a cross of wheat cultivars shanghai 3/Catbird and naxos. Theor. Appl. Genet. 127, 1873–1883. doi: 10.1007/S00122-014-2346-3/TABLES/3 24970343

[B250] Zhou X. L. Wang M. N. Chen X. M. Lu Y. Kang Z. S. Jing J. X. (2014). Identification of Yr59 conferring high-temperature adult-plant resistance to stripe rust in wheat germplasm PI 178759. Theor. Appl. Genet. 127, 935–945. doi: 10.1007/s00122-014-2269-z 24487945

[B251] Zou J. Semagn K. Chen H. Iqbal M. Asif M. N’Diaye A.. (2017). Mapping of QTLs associated with resistance to common bunt, tan spot, leaf rust, and stripe rust in a spring wheat population. Mol. Breed. 37, 1.14. doi: 10.1007/S11032-017-0746-1 28127252

[B252] Zwart R. S. Thompson J. P. Godwin I. D. (2005). Identification of quantitative trait loci for resistance to two species of root-lesion nematode (*Pratylenchus thornei* and *P. neglectus*) in wheat. Aust. J. Agric. Res. 56, 345–352. doi: 10.1071/AR04223

[B253] Zwart R. S. Thompson J. P. Milgate A. W. Bansal U. K. Williamson P. M. Raman H.. (2010). QTL mapping of multiple foliar disease and root-lesion nematode resistances in wheat. Mol. Breed. 26, 107–124. doi: 10.1007/s11032-009-9381-9

